# A novel approach for sampling plant mass-density relationships

**DOI:** 10.1016/j.fmre.2025.06.009

**Published:** 2025-06-29

**Authors:** Liang Zhang, Shubin Xie, Renfei Chen, Jinzhi Ran, Jianming Deng

**Affiliations:** a State Key Laboratory of Herbage Improvement and Grassland Agro-ecosystems, College of Ecology, Lanzhou University, Lanzhou 730000, China; b School of Life Science, Shanxi Normal University, Taiyuan 030000, China

**Keywords:** Quadrat sampling, Mass-density relationship, Voronoi diagram, Random sampling, Simulation, Large plot

## Abstract

In plant ecology, traditional quadrat-based methods are widely employed to investigate plant mass-density relationships. However, this approach has limitations when applied to irregularly distributed plant populations and communities. To address this challenge, we developed a novel sampling method based on Voronoi diagrams, designed to improve field investigations of mass-density relationships. The Voronoi diagram-based sampling approach defines irregular polygonal plots by merging the Voronoi cells of multiple individuals around a sampling point, enabling more precise delineation of the area and boundaries occupied by plants. To demonstrate the effectiveness and advantages of the Voronoi diagram-based method, we compare it with the traditional quadrat-based method in accessing the classic plant mass-density relationship, which has been suggested to follow a general scaling law. Here the novel approach demonstrated superior performance, providing a more precise and reliable analysis of the plant mass-density relationship. Moreover, our results reveal that the scaling exponent of average plant biomass to population density increases rapidly and then stabilizes near -4/3 as the number of individuals per plot (NI) exceeds 50, driven by declining variability in both average plant biomass and density. This generalizable approach, independent of individual spatial patterns, facilitates efficient empirical data collection with relatively fewer individuals, offering practical advantages for field-based studies. This approach provides a robust and precise tool for investigating and analyzing complex ecosystems, with broad applications in forest biomass estimation, plant interaction modeling, and even large-scale ecological analysis using remote sensing data. It thus holds significant potential for advancing ecological research and guiding ecosystem monitoring and management.

## Graphical abstract

**Figure:**
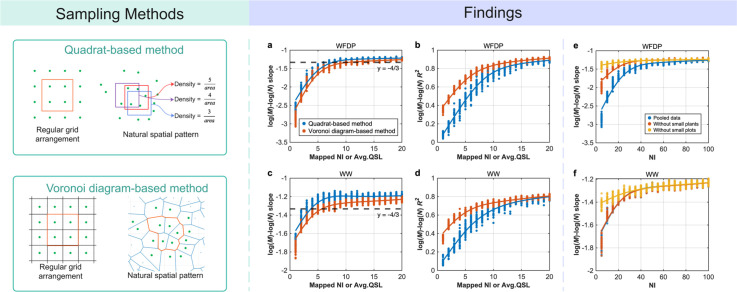

## 1 Introduction

In plant ecology, a widely accepted approach is used to understand ecological phenomena and test theoretical laws, that is, selecting several representative plant individuals denoting the whole population or community, i.e., “sampling”. As a basic and common procedure in ecological studies across various goals, empirical data derived from sampling is indispensable because it provides foundational information for subsequent data analyses. Therefore, whether we sample suitable plant individuals would directly influence the correction of the scientific results and conclusions drawn. So far, the widely accepted sampling approach is plotting with regular shapes, such as squares [[1], [2], [3]] and circles [4]. A common type of plant data involves plant size (e.g., height, diameter at breast height), species richness, and the number of each species within each forest quadrat. However, this classical quadrat-based sampling approach has limitations because it is more effective in flat terrains where plant populations and communities are spatially uniform, such as in density-controlled experiments. In such conditions, plants are typically distributed at regular intervals, and each individual occupies the same amount of space. By contrast, in natural ecosystems, there are generally three types of spatial patterns: uniform, random, and clustered [5,6]. The random and clustered types are usually irregular distribution patterns, resulting in individual plants occupying variable amounts of space. Therefore, this quadrat-based sampling method may yield imprecise estimates of plant and community attributes, such as average biomass per plant and abundance, and thus their correlations, especially when spatial distributions of vegetation are random or aggregated.

To address this issue, we developed a Voronoi diagram-based sampling approach tailored to capture plant mass-density relationships in natural systems with various spatial distribution patterns. The mass-density scaling relationship describes how average plant biomass varies with population density, reflecting the trade-off between individual size and abundance within plant communities. This relationship is critical for understanding and predicting plant population or community dynamics and their productivities, which can reflect both the dynamics of individual plant growth and community structure, and thus has been widely used across diverse ecosystems, including deserts [7], grasslands [3,8], forests [9,10], intertidal zones [1], agroecosystems [11,12], and urban woodlands [13]. Yoda used a geometric similarity model and the assumption of constant resource availability per area to derive the −3/2 self-thinning exponent, in terms of the relationship between mean biomass per plant and population density [14]. Enquist et al., [15] based on the assumption that individual resource use scales approximately with the 3/4 power of body mass and total energy use remains constant, predicted that average plant size scales as the −4/3 power of maximum population density. Although self-thinning describes temporal population dynamics within a stand, most mass-density relationships are frequently inferred from spatial snapshots, potentially integrating multiple successional stages or environmental conditions [16]. Prior research has identified that this relationship is often influenced by a range of environmental and biological factors, including light availability [17], soil fertility [18,19], salinity [1], water availability [4,7,20], and species-specific traits [21,22]. Experimental and modeling studies have further highlighted the roles of competition [23,24], biomass allocation patterns [25], and types of interaction [24,26,27] in shaping the scaling exponent. In studies investigating plant biomass-density relationships using quadrat-based sampling methods, quadrats of varying sizes are frequently employed; however, the influence of quadrat size on the derived relationships remains largely unexplored [1,4,7]. Size inequality weakens the mass-density relationship by changing the self-thinning pattern [28]. To summarize, variations in environmental conditions, species traits, quadrat sizes, and size inequality among individuals all affect the estimation of mass-density scaling relationships. Acknowledging and accounting for these factors is essential for improving the reliability and generalizability of such analyses.

The Voronoi diagram is a geometric structure that partitions two-dimensional space into non-overlapping regions, each encompassing all locations closest to a given point [29]. It delineates the spatial domain associated with each point, capturing its nearest neighborhood. Typically constructed via Delaunay triangulation, the diagram is formed by drawing perpendicular bisectors of triangle edges, whose intersections define the Voronoi boundaries. This method enables the geometric subdivision of space among adjacent individuals. Voronoi diagrams also have several important applications in ecology. For example, they are used to delineate the potential area of influence around individual plants, enabling researchers to quantify competitive interactions based on spatial proximity [30]. In forest ecology, weighted Voronoi diagrams incorporate size-related attributes such as diameter or height to simulate asymmetric competition [31]. Furthermore, in ecological monitoring using autonomous systems, Voronoi partitioning enables efficient allocation of survey areas among unmanned aerial vehicles, reducing overlap and improving search efficiency [32]. These applications demonstrate the versatility of Voronoi diagrams in linking spatial structure with ecological processes, from individual-level competition to landscape-scale management. However, it remains unclear whether Voronoi diagrams provide substantial advantages in ecological investigation and accurate analysis over traditional sampling methods, beyond facilitating investigations of irregular spatial distributions.

To address the gap in evaluating the advantages of Voronoi diagram-based sampling in ecological investigation and analysis, we first applied Voronoi diagrams to more precisely estimate the space occupied by each individual and define spatial boundaries [29], which were then used to establish the boundaries of sampling units. Subsequently, by merging the Voronoi cells of varying numbers of individuals (NI) around each sampling point, we generated irregular polygons as sampling plots. Thirdly, using comprehensive datasets from Wabikon Forest Dynamics Plot (WFDP) and Wytham Woods (WW), we conducted random sampling simulations with different NI values as sampling conditions. We then fitted the resulting log(average biomass) (i.e., log*M*) and log(population/community density) (i.e., log*N*) data to obtain the scaling exponent (slope), intercept, and corresponding *R*^2^ of the mass-density relationship. Finally, compared to the traditional square quadrat-based sampling method, our proposed Voronoi diagram-based method demonstrated superior performance in analyzing the plant mass-density relationship, offering a more reliable depiction of biomass-density dynamics. The mass-density (M-N) relationship supports a broad spectrum of ecological and management applications, including vegetation management, agricultural optimization, biodiversity conservation, biomass estimation, and ecosystem modeling [11,21,[33], [34], [35]]. Building on this foundation, our study further examines the potential of the proposed method to improve forest biomass estimation and guide density management in experimental design.

## 2 Materials and methods

### 2.1 From quadrat-based to Voronoi diagram-based sampling methods

**Fig. 1:**
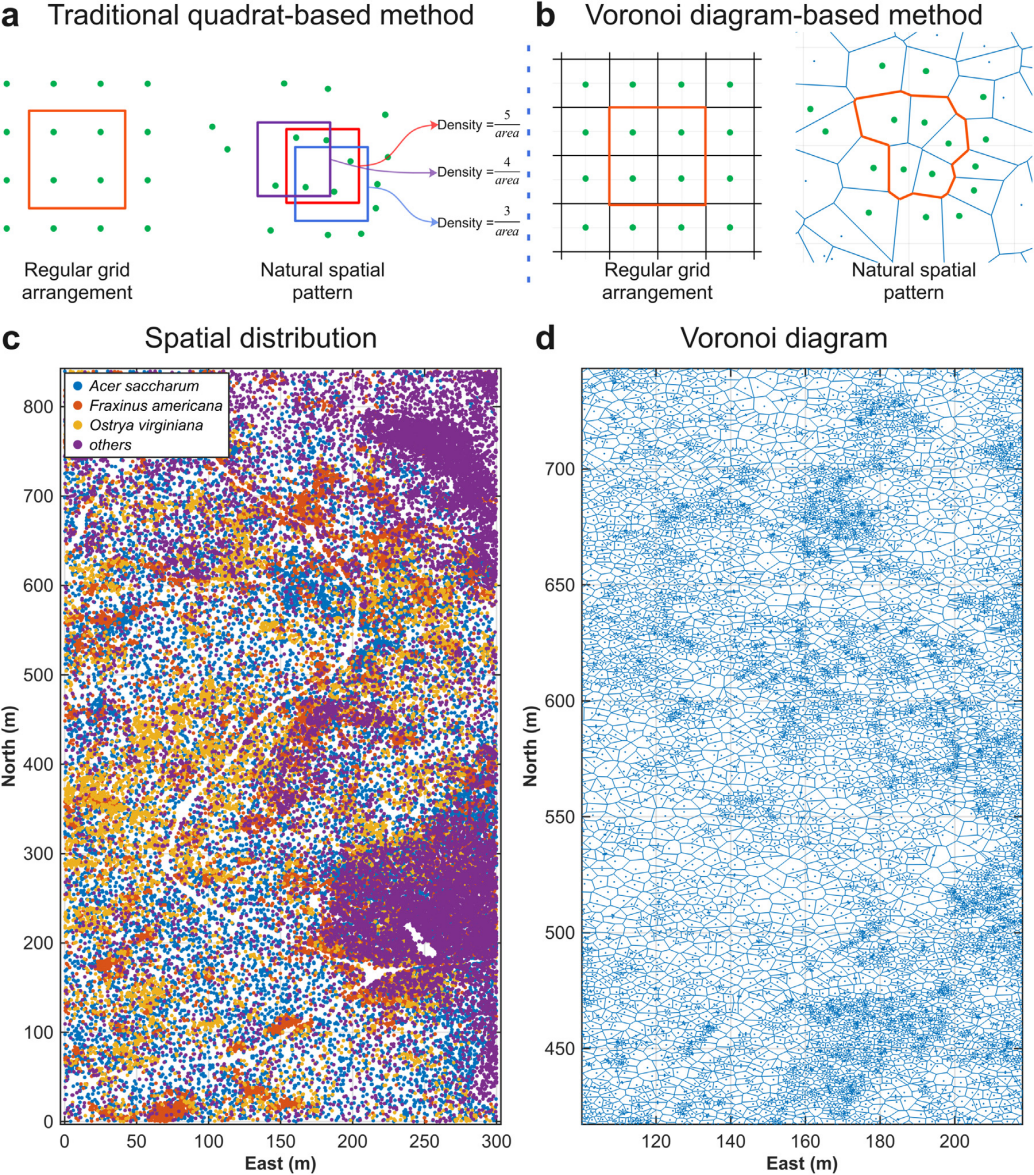
**Overview of sampling methods, spatial distribution, and Voronoi diagram for part Wabikon Forest Dynamics Plot (WFDP)**. (a) Illustration of the traditional square quadrat-based sampling method. (b) Overview of the Voronoi diagram-based sampling method, with sampling plots highlighted in orange. (c) Spatial distribution of the three most abundant species in WFDP, represented by points in three different colors. (d) Voronoi diagram partitioning for each plant in a part of WFDP.

In sampling plots where plant spatial distribution is non-uniform, the number of plant individuals varies depending on the spatial location (Fig. 1a). As a result, the observed mass-density relationships may be different when plant individuals are sampled at different locations, which introduces substantial uncertainty into the scientific conclusions.

To overcome the limitations, we developed a new sampling method tailored for investigating mass-density relationships. Specifically, we first built Voronoi diagrams to spatially partition individual plants within a sampling plot, where each Voronoi cell represents the spatial domain occupied by a single plant [29]. By fixing the number of individuals per plot and merging multiple adjacent Voronoi cells, we derive an irregularly shaped polygon as the sampling plot (Fig. 1b). To the end, the Voronoi diagram-based method represents a multi-scale sampling approach by maintaining a fixed number of plant individuals per sampling plot while allowing the plot areas to vary. This stands in contrast to the quadrat-based method, which commonly involves collecting a variable number of individuals within a fixed-area plot. This spatial partitioning method not only allows for the visualization and calculation of the space occupied by each individual but also enables the convenient merging of spaces occupied by multiple individuals.

### 2.2 Empirical dataset

To show the advantages of the Voronoi diagram-based sampling method, we perform empirical analyses with the dataset from the Wabikon Forest Dynamics Plot (WFDP). The WFDP is located in the Chequamegon-Nicolet National Forest’s Lakewood-Laona District in northeastern Wisconsin, USA (N45.5546°, W88.7945°). This temperate continental forest features a rectangular plot of 840 m × 300 m, with the census data in 2008–09 used in our analysis. The plot contained 48,835 individuals from 36 species, dominated by *Acer saccharum* (31.87%), *Fraxinus americana* (19.25%), and *Ostrya virginiana* (15.64%). The Wytham Woods (WW), a mixed deciduous forest in southern England (N51.7743°, W1.3379°), covers approximately 400 hectares as a temperate oceanic forest, making it a substantial woodland area for the United Kingdom. The core area of WW, measuring 300 m × 600 m, was censused in 2010 [36]. This plot contains 15,895 individuals from 23 species, with *Acer pseudoplatanus* (47.39%) and *Fraxinus excelsior* (32.68%) being the dominant species. These datasets include detailed information on each individual’s location, diameter at breast height, and species name. The spatial distribution of each individual within the WFDP is illustrated in Fig. 1c, while that for WW is presented in Fig. S1, where the three most abundant species are marked with distinct colors. This dataset can be requested for reasonable usage from the Forest Global Earth Observatory (ForestGEO) http://ctfs.si.edu/datarequest/.

### 2.3 Empirical data analyses

For WFDP and WW, the biomass of each individual is calculated using the “allodb” R package, designed for biomass estimation at globally distributed extratropical forest plots [37]. This package incorporates systematically selected published allometric equations and proposed functions to compute aboveground biomass, based on data from 701 woody species identified across 24 ForestGEO (including WFDP and WW) forest dynamics plots. The recorded diameter at breast height, species name, and the geographic coordinates of the plots are utilized by “allodb” to generate the biomass estimates. By first estimating the biomass of each surviving stem within the WFDP and WW, and then aggregating these values for each plant, we derived the total biomass at the individual level. The WFDP and WW data were then organized to include spatial positions and individual biomass, preparing them for further analysis.

The construction of the Voronoi diagram is based on the coordinate positions of the plants, with the boundaries of each cell determined by the perpendicular bisectors between pairs of neighboring plants, effectively dividing the entire space into several non-overlapping regions [29]. In a Voronoi diagram, all points within a given cell are closer to the plant at the center (i.e., the generating point) than to any other plant. Thus, each Voronoi cell can be reasonably interpreted as the “occupied space” of the corresponding plant, reflecting its spatial resource competition and influence within its environment. Accurate spatial coordinates of each individual within the full plot are a prerequisite for constructing Voronoi diagrams in both the WFDP and WW sites, as they determine the geometry of the resulting spatial partitions.

The spatial partitioning of the WFDP plot using Voronoi diagrams [29] is partially illustrated in Fig. 1d, while that for WW is presented in Fig. S1b. Individuals within 5 m of the plot boundary were excluded from the analysis, as the Voronoi polygons at the edges could become disproportionately large or irregular in shape due to the absence of neighboring plants. The remaining 46,339 individuals from the WFDP and 15,223 from WW were used for the mass-density relationship study.

### 2.4 Modeling mass-density relationship

After obtaining a set of plot-level data, we first calculated the mean biomass and density for each plot. These two variables are generally assumed to follow an allometric (power-law) relationship of the form:

(1) $$M=K\times N^{b}$$

where *M* is the average biomass per plant, *N* is the density (number of individuals per unit area), *K* is a constant, and *b* is the scaling exponent. Fitting this nonlinear exponential relationship directly can be computationally complex. To simplify the analysis, we applied a logarithmic transformation to both sides of the equation, resulting in a linear form:

(2) $$\begin{array}{c} \log M=\log K+b\times \text{log}N \end{array}$$

There is a clear linear relationship between log*M* and log*N*, where log*K* and b represent the intercept and slope, respectively. Therefore, in our analysis of the mass-density relationship, we performed linear regression on the log-transformed variables and referred to the slope *b* as the scaling exponent.

Given that both *M* and *N* are subject to measurement errors in plot-based sampling, we adopted Reduced Major Axis (RMA) regression [38] to fit their relationship, as it accounts for errors in both the dependent and independent variables. Unlike ordinary least squares regression, which attributes error solely to the dependent variable, RMA is more suitable when both variables are field-derived and error-prone. In our study, biomass and density were calculated from independently measured individual-level diameter and spatial coordinates, both of which are subject to inherent inaccuracies. All variables were log-transformed using natural logarithms before analysis. It is important to note that the WFDP plot contains 36 species, whereas the WW plot contains 23 species, with each sampling plot possibly containing *multiple species*. Nevertheless, we use the term “density” to refer to the number of individuals per unit area.

### 2.5 Simulation of random sampling

Based on the prepared WFDP and WW datasets, which include the spatial position, biomass of each individual, and the area calculated using the Voronoi diagram, we conducted random sampling simulations to study the mass-density relationship at different scales. Instead of using a quadrat-based sampling method, we selected a specific number of individuals, with the irregular polygons formed by their Voronoi cells serving as sampling plots (Fig. 1b). The simulations were performed using varying numbers of individuals (NI, ${\left\{s_{i}\right\}}_{i=1}^{n_{1}}$) and a fixed number of sample plots ($n_{2}$). Each simulation was independently executed $n_{3}$ times to ensure robustness.

The simulation begins by generating $n_{2}$ pairs of uniformly distributed random coordinates within the boundaries of the large plot, which serve as the initial location coordinates. For each set of coordinates, we calculate the distance to each individual and select the nearest $s_{i}$ individuals. This ensures that the Voronoi cells of the $s_{i}$ individuals are adjacent and can be merged into a continuous area of space. The Voronoi cells of these $s_{i}$​ individuals are then merged to form an irregular polygon as the sampling plot. The plot boundary is delineated by the merged Voronoi cells of the $s_{i}$ sampled individuals, with the outer perimeter formed by the connected edges of the Voronoi tessellation, as shown in Fig. 1b. In this context, a series of connected line segments fairly delineated the space occupied by plant individuals inside and outside the sampling plot. Subsequently, the log*M*-log*N* data are then computed and fitted via RMA regression [38].

This approach yields a set of parameters for each simulation: a scaling exponent, an intercept, and a corresponding *R*^2^ value. Ultimately, we obtain $n_{1}\times n_{2}\times n_{3}$ simulation results, which are further analyzed to examine how the scaling exponent, intercept, and *R*^2^ vary with the NI (number of individuals per plot). Our simulation methodology is outlined in Algorithm 1 and was implemented using MATLAB. In the simulation, we set the number of repetitions ($n_{3}$) to 30 and the number of plots ($n_{2}$) to 1000. This repetition count was chosen to ensure the robustness and stability of the results and serves as the threshold value for statistical analyses [39,40]. The NI (${\left\{s_{i}\right\}}_{i=1}^{n_{1}}$) ranges from 5 to 100, increasing incrementally by 5, with $n_{1}$ equal to 20. We conducted simulations at NI = 5, 50, and 100 using varying numbers of replicates. The results revealed that the coefficient of variation (CV) of the log*M*-log*N* slope increased initially with the number of replicates and then gradually leveled off (Fig. S2). Notably, the CV reached relatively high and stable values when the number of replicates was around 30, indicating that this replicate number provides a reasonable balance between computational efficiency and result stability.

**Algorithm 1:** Simulation of random sampling.

Initialization: large plot dataset A, $n_{1}$ number of individuals per plot ${\left\{s_{i}\right\}}_{i=1}^{n_{1}}$, number of plots $n_{2}$, number of repetitions $n_{3}$.
for $i=1\to n_{1}$ do
for $j=1\to n_{3}$ do
Generate $n_{2}$ uniformly distributed random coordinates within A;
Calculate the distance to each individual and select the nearest $s_{i}$ individuals;
Calculate the log*M* and log*N* for each plot;
Fit log*M* vs. log*N* with reduced major axis regression;
Store the slope, intercept, and *R*^2^.
end for
end for

We model the trends in the fitted slope, intercept, and *R*^2^ values as NI varies using Generalized Additive Models (GAMs) with the “gam” package in R [41]. To identify the breakpoint in the changing pattern of slope, intercept, and *R*^2^ with increasing NI, we applied segmented regression—focusing solely on changes in slope—across continuous thresholds using the “chngpt” package in R [42]. The slope, intercept, and *R*^2^ of the log(average biomass) vs. log(density) relationship exhibit variability with NI, especially when NI is small. To quantify this variability, we first determine a “threshold” that represents the breakpoint in the changing pattern. We then select all data points with NI that are no bigger than this threshold and perform a linear regression of these data against NI. The resulting fitted slope serves as a measure of variability, with smaller slopes indicating lower variability.

### 2.6 Quadrat-based method for comparison

Traditional square quadrat-based sampling is utilized for comparison with our proposed method. As shown in Fig. 3e–f, the sampled plot area under different NI (number of individuals per plot) conditions approximates a normal distribution. To ensure a fair comparison, the quadrat-based method employed a combination of multiple quadrat side lengths for sampling. Under each simulation condition with the same NI, we aggregated the sampled plot area data from 30 repeated simulations, constructed a frequency distribution histogram with 100 intervals, and proportionally mapped these frequencies to integers summing to 1000 to maintain consistency in the number of sampling plots. The mean value of each interval was used as the quadrat area, while the mapped frequencies represented the corresponding number of square quadrats. During the actual calculation process, certain sampling conditions, defined by quadrat area and number, yielded fewer than 100 sets due to the lower probability of occurrence within specific intervals. Random points were uniformly distributed to define the lower-left corners of square quadrats, with their range constrained to avoid edge effects and ensure full containment within the larger plot. Except for the shape of the quadrats being square, the remaining procedure was consistent with our sampling simulation (Algorithm 1). We used the average quadrat side length (Avg. QSL) of each simulation to label different sampling conditions of the quadrat-based method, corresponding to NI. Additionally, for ease of comparison, we mapped NI and Avg. QSL to integers ranging from 1 to 20.

At larger spatial scales, we generated random points across the full extent of the large plot to serve as square plot centers. To minimize edge constraints, plots near the boundary were adjusted to remain entirely within the study area by aligning them with the nearest edge. Experiments were conducted with NI ranging from 25 to 500 in steps of 25, and the results are presented in Fig. S3. The results demonstrate that the Voronoi diagram-based method outperforms the quadrat-based method, as evidenced by consistently higher *R*^2^ values. Nevertheless, this performance gap narrows with increasing spatial scale, becoming nearly absent in the WFDP plot and marginal in the WW plot.

In field-based studies of the plant mass-density relationship, square quadrats are most commonly used [[1], [2], [3]], although some studies have employed circular plots [4]. To evaluate the effect of plot shape on sampling outcomes, we conducted a systematic comparison between square and circular plots of equal area. The results showed that while the slopes of the log*M*-log*N* relationships were generally similar between the two plot shapes, intercepts exhibited larger differences, and the coefficient of determination (*R*^2^) was slightly higher when using circular plots (Fig. S4). We further compared the differences in mean values under various conditions. In the WFDP plot, the largest difference in *R*^2^ occurred at a mapped Avg. QSL of 5, reaching 0.12, followed by differences of 0.10 and 0.11 at Avg. QSL values of 4 and 6, respectively; under all other conditions, the differences were < 0.1, with the smallest value being 0.021 at Avg. QSL = 18. Similarly, in the WW plot, the largest difference was observed at Avg. QSL = 5 (0.08), while the smallest was at Avg. QSL = 1 (0.025). Owing to the minor differences observed in the *R*^2^ values of log*M*-log*N* relationships between circular and square plots, and considering the widespread use of square plots in mass-density relationship studies, we adopted square plots as the basis for comparison with the Voronoi diagram-based method.

To determine an appropriate interval setting, we compared the simulation results of the quadrat-based method using histograms divided into 50, 100, and 200 intervals. The outcomes across these different interval settings were highly consistent, with only negligible differences observed (Fig. S5). Based on this, we selected 100 intervals as a balanced choice that ensures both the robustness and reliability of the results while keeping the computational workload at a manageable level.

### 2.7 Effects analysis of small plants and plots

To study the effect of small plants and small plots on the utility of our Voronoi diagram-based sampling approach, we define several thresholds to assess the variations of the scaling exponent of plant mass-density relationships under two scenarios: (1) individuals with biomass < 1 kg were excluded (using individuals with biomass > 1 kg for simulation) and (2) plots with log(average biomass) less than a threshold were excluded (using all the individuals for simulation followed by fitting plot data with high value of log(average biomass)).

In scenario (1), we empirically selected plant individuals with biomass > 1 kg as large plants, filtering 27,474 (occupying 59.29%) individuals from the WFDP and 14,971 (occupying 98.34%) individuals from the WW dataset. Voronoi diagrams [29] were then used to calculate their occupied area. Then, we conducted simulations with Algorithm 1 to observe how the scaling exponent of log (average biomass)-log (density) changes with NI.

In scenario (2), we perform similar simulations with all the plant individuals under each dataset. For WFDP, we used K-means (*k* = 2) clustering [43] to separate the main distribution from the outlier distribution of average biomass at NI = 5, taking the midpoint between the two cluster centers as the dividing point. We calculated the dividing point for the 30 repeated simulation results and used the mean value as the final dividing point. This dividing point was identified as log (average biomass) = 2.83 and was used to filter data across all NI conditions, retaining only log(average biomass) > 2.83 for WFDP. For the WW dataset, we empirically defined plots with log (average biomass) > 4 as large plots, based on the distribution of average biomass (Fig. 3d). We then refitted the relationship between log (average biomass) and log(density) using Reduced Major Axis regression [38].

To enable a more detailed comparison of sampling outcomes under different scenarios, we introduced two statistical measures: the coefficient of variation (CV) and the Gini coefficient [44]. Specifically, we calculated the CV of log(*M*) and log(*N*) across all plots within each sampling process under varying NI conditions, thereby revealing how the variability of these parameters changes with NI. In addition, for each plot obtained under a given NI condition, we computed the Gini coefficient based on individual biomass values within the plot, as a measure of size inequality. The mean Gini coefficient for each independent sampling process was then used to characterize the trend of biomass inequality to NI. The Gini coefficient, a measure of inequality, can be calculated from a given dataset $\left\{x_{1},x_{2},\ldots ,x_{n}\right\}$ (sorted in ascending order) using the formula:

(3) $$G=\frac{2\sum_{i=1}^{n}ix_{i}}{n\sum_{i=1}^{n}x_{i}}-\frac{n+1}{n}$$

where $x_{i}$ represents the value of the *i* th individual, and *n* is the total number of observations.

### 2.8 Predict forest biomass

Given the fitted slope *a_1_* and intercept *b_1_* of the log*M*-log*N* relationship ($\text{log}M=b_{1}+a_{1}\times \text{log}N$), along with measurements of the overall forest density *N* and area, the total biomass of the forest can be predicted using the formula: $e^{b_{1}}\times N^{a_{1}+1}\times area$. Additionally, by partitioning the spatial distribution using Voronoi diagrams [29], the density and area of each individual can be determined, allowing for the prediction of individual biomass and, subsequently, the total biomass. This approach will be employed as our method for predicting the total biomass of the forest plots. In parallel, for comparative purposes, we estimated the total biomass based on the overall area and density of the large plot.

Since repeated simulations were conducted under each sampling condition, the mean slopes and intercepts from these repetitions were used to estimate the total biomass. Furthermore, as scenario 2 (using all the individuals for simulation followed by fitting plot data with a high value of log (average biomass)) exhibited the smallest variability in the slope and intercept of the log*M*-log*N* relationship across different NI values (Fig. 5a–f), we utilized the simulation results from this scenario to predict biomass. Finally, the predicted biomass was compared to the total biomass calculated by summing individual biomass values determined using the “allodb” package [37]. This comparison was conducted to evaluate the validity and reliability of our biomass estimation method. In the results, we present the biomass prediction corresponding to the NI condition that achieved the best performance.

## 3 Results

**Fig. 2:**
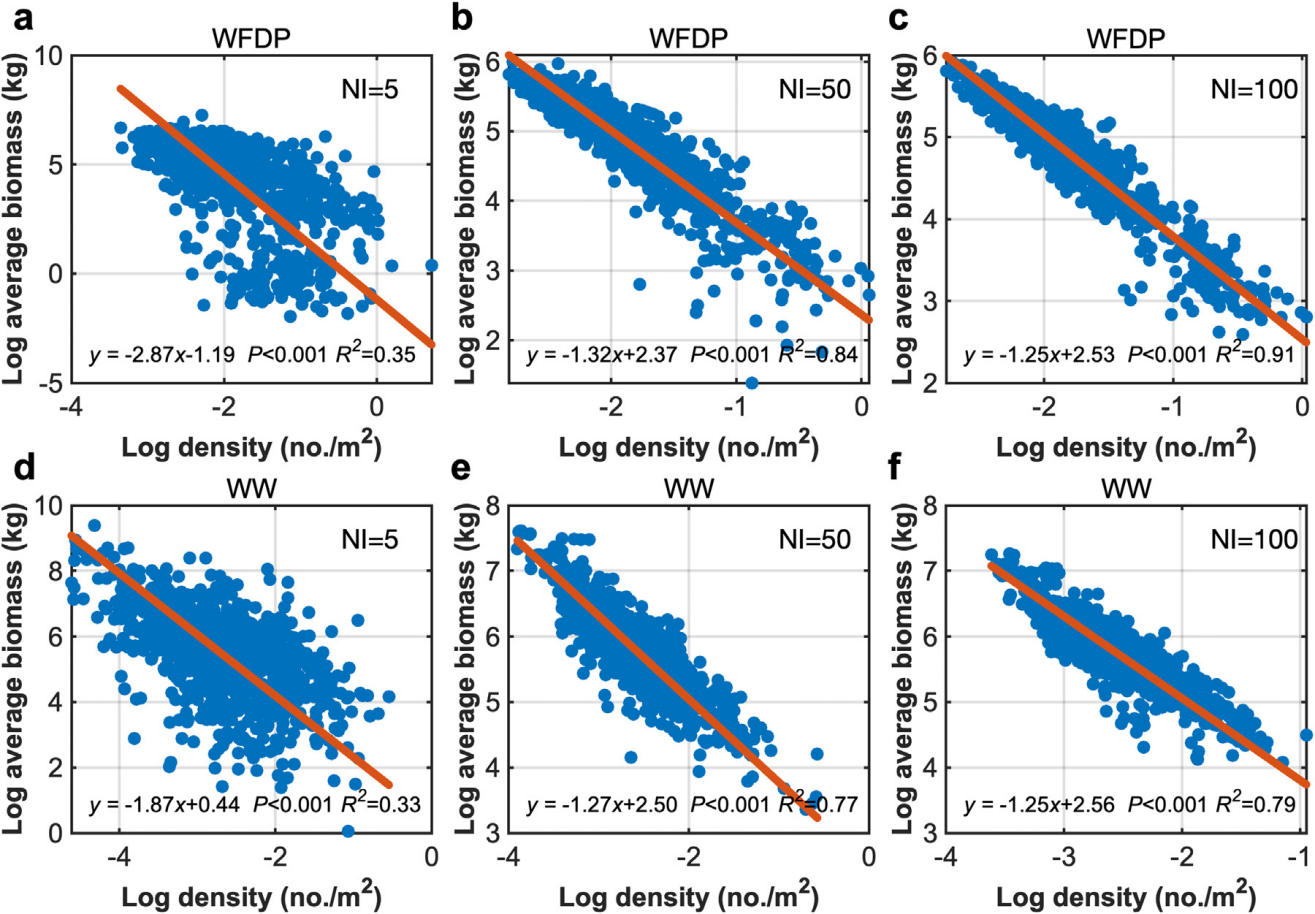
**The linear relationship between log(average biomass) and log(density) under the Voronoi diagram-based sampling method**. Statistical regression is performed with the Reduced Major Axis (RMA) regression method. Each figure specifies the dataset and the number of individuals per plot (NI) used in the simulation, along with the RMA-fitted function. All fitted results are significant at the *P* < 0.001 level.

The performance of the Voronoi diagram-based method improves with increasing NI. As the number of individuals per sampled plot (NI) increases, the scaling relationship between plant average biomass (*M*) and density (*N*) becomes more significant, with a notable improvement in the model fit (Figs. 2 and S6, S7). When NI is small, a considerable number of data exhibit substantial dispersion, leading to a poor model fit (e.g., *R*^2^ = 0.35 when NI = 5 at WFDP, *R*^2^ = 0.33 when NI = 5 at WW). On the contrary, when NI is large, the data points progressively converge around the regression line, which significantly enhances the model fit (e.g., *R*^2^ = 0.91 when NI = 100 at WFDP, *R*^2^ = 0.79 when NI = 100 at WW; Fig. 2).

**Fig. 3:**
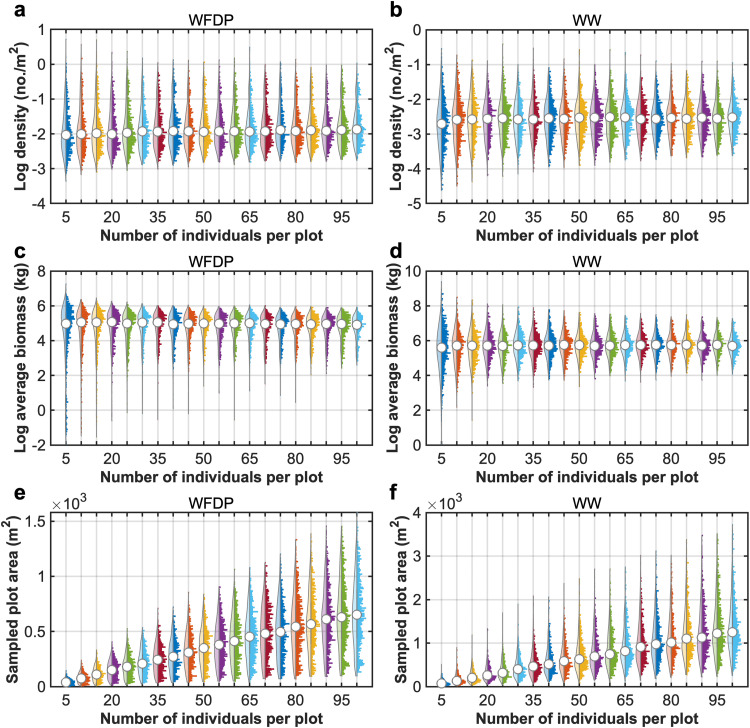
**Simulation results of the Voronoi diagram-based sampling method**. (a, b) The distributions of log(density) (log*N*) obtained from simulations under different NI (number of individuals per plot) values using the Voronoi diagram-based method, illustrated using both probability density functions and histograms. (c, d) The distributions of log(average biomass) (log*M*) obtained using the Voronoi diagram-based method. (e, f) The distributions of the sampled plot area generated through the Voronoi diagram-based method.

**Fig. 4:**
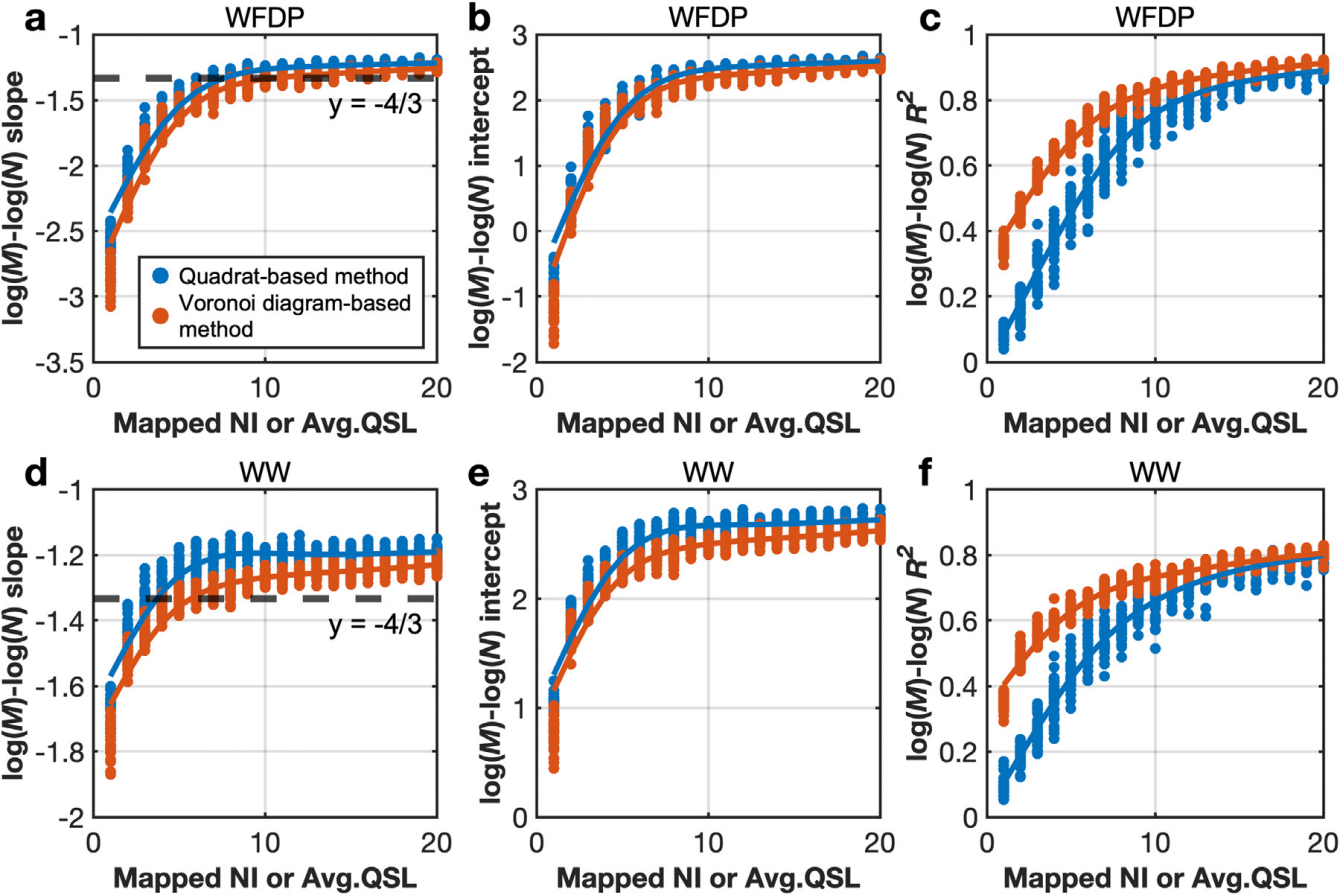
**Comparisons of simulation results between the Voronoi diagram-based method and the quadrat-based method**. Comparisons of the slopes (a, d), intercepts (b, e), and corresponding *R^2^* values (c, f) of the log(average biomass)-log(density) (log*M*-log*N*) relationship fitted using the Reduced Major Axis regression for the Voronoi diagram-based method and the quadrat-based method are illustrated. Red points represent simulation results using the Voronoi diagram-based method, while blue points represent results from the quadrat-based method. Each point corresponds to a single simulation, and the solid lines represent the trend fitted using Generalized Additive Models. All RMA-fitted results are significant at the *P* < 0.001 level.

The new Voronoi diagram-based sampling method outperformed the traditional quadrat-based approach. As NI increases, plant density shows limited variation with a small magnitude of change, while plant average biomass exhibits a broader range of variation, and both the range and mean values of the sampled plot area increase (Figs. 3 and S8). The number of plots with small average biomass decreases when NI decreases (Figs. 2, 3c–d). For the scaling relationship between average biomass (*M*) and density (*N*) under both the Voronoi diagram-based method and the quadrat-based method, the slope, intercept, and corresponding *R*^2^ value initially increase rapidly with increasing NI or average quadrat side length (Avg.QSL) and then stabilize (Fig. 4; Table 1, Table 2). The threshold for the two changing trends in both the slope and intercept occurs at mapped NI = 4 at WFDP and 3 at WW, while the threshold for *R*^2^ is mapped NI = 6 at WFDP and 5 at WW. Using the quadrat-based method, the slope and intercept of the log(average biomass)-log(density) (log*M*-log*N*) relationship are slightly higher than those of the Voronoi diagram-based method, whereas the *R*^2^ value corresponding to the Voronoi diagram-based method is larger than that of the quadrat-based method (Fig. 4). Additionally, the Voronoi diagram-based method exhibits a smaller standard deviation in its *R*^2^ values compared to the quadrat-based method (Tables 1, 2).

**Table 1:** **Simulation results of the Voronoi diagram-based method and the quadrat-based method at Wabikon Forest Dynamics Plot (WFDP)**.

Mapped NI or Avg.QSL	Voronoi diagram-based method		Quadrat-based method	
	Slope	*R*^2^	Slope	*R*^2^
1	−2.85 ± 0.10	0.35 ± 0.02	−2.57 ± 0.08	0.08 ± 0.02
2	−2.24 ± 0.07	0.46 ± 0.02	−2.05± 0.09	0.17 ± 0.04
3	−1.91 ± 0.09	0.56 ± 0.02	−1.77 ± 0.09	0.27 ± 0.06
4	−1.71 ± 0.07	0.63 ± 0.03	−1.63 ± 0.08	0.36 ± 0.06
5	−1.59 ± 0.05	0.68 ± 0.02	−1.48 ± 0.05	0.47 ± 0.05
6	−1.50 ± 0.05	0.73 ± 0.02	−1.45 ± 0.06	0.52 ± 0.06
7	−1.47 ± 0.05	0.76 ± 0.02	−1.36 ± 0.04	0.62 ± 0.06
8	−1.40 ± 0.04	0.80 ± 0.02	−1.32 ± 0.04	0.70 ± 0.06
9	−1.39 ± 0.04	0.81 ± 0.02	−1.30 ± 0.04	0.73 ± 0.05
10	−1.35 ± 0.03	0.83 ± 0.01	−1.28 ± 0.03	0.76 ± 0.04
11	−1.34 ± 0.03	0.85 ± 0.01	−1.26 ± 0.02	0.79 ± 0.03
12	−1.33 ± 0.03	0.86 ± 0.01	−1.26 ± 0.02	0.80 ± 0.03
13	−1.31 ± 0.02	0.87 ± 0.01	−1.25 ± 0.02	0.83 ± 0.03
14	−1.30 ± 0.02	0.88 ± 0.01	−1.24 ± 0.02	0.84 ± 0.02
15	−1.29 ± 0.02	0.89 ± 0.01	−1.23 ± 0.02	0.85 ± 0.02
16	−1.28 ± 0.02	0.89 ± 0.01	−1.23 ± 0.02	0.87 ± 0.01
17	−1.28 ± 0.02	0.90 ± 0.01	−1.22 ± 0.02	0.87 ± 0.01
18	−1.27 ± 0.02	0.90 ± 0.01	−1.23 ± 0.01	0.88 ± 0.01
19	−1.27 ± 0.02	0.91 ± 0.01	−1.22 ± 0.02	0.88 ± 0.01
20	−1.26 ± 0.02	0.91 ± 0.01	−1.22 ± 0.01	0.89 ± 0.01

Each reported value represents the average of 30 independent simulations, with the corresponding standard deviation provided.

**Table 2:** **Simulation results of the Voronoi diagram-based method and the quadrat-based method at Wytham Woods (WW)**.

Mapped NI or Avg. QSL	Voronoi diagram-based method		Quadrat-based method	
	Slope	*R*^2^	Slope	*R*^2^
1	−1.76 ± 0.05	0.35 ± 0.02	−1.67 ± 0.04	0.09 ± 0.03
2	−1.51 ± 0.03	0.49 ± 0.03	−1.43 ± 0.04	0.18 ± 0.03
3	−1.43 ± 0.03	0.56 ± 0.03	−1.33 ± 0.05	0.27 ± 0.05
4	−1.37 ± 0.02	0.60 ± 0.02	−1.28 ± 0.03	0.36 ± 0.06
5	−1.34 ± 0.02	0.64 ± 0.02	−1.24 ± 0.03	0.44 ± 0.05
6	−1.32 ± 0.02	0.66 ± 0.02	−1.23 ± 0.03	0.51 ± 0.05
7	−1.31 ± 0.02	0.69 ± 0.02	−1.22 ± 0.03	0.55 ± 0.04
8	−1.30 ± 0.03	0.71 ± 0.02	−1.21 ± 0.04	0.59 ± 0.04
9	−1.28 ± 0.02	0.72 ± 0.02	−1.19 ± 0.03	0.63 ± 0.03
10	−1.27 ± 0.02	0.73 ± 0.02	−1.21 ± 0.02	0.65 ± 0.05
11	−1.27 ± 0.02	0.74 ± 0.02	−1.20 ± 0.02	0.69 ± 0.03
12	−1.26 ± 0.02	0.75 ± 0.01	−1.19 ± 0.02	0.71 ± 0.03
13	−1.25 ± 0.02	0.76 ± 0.02	−1.21 ± 0.02	0.72 ± 0.03
14	−1.25 ± 0.02	0.77 ± 0.01	−1.20 ± 0.02	0.74 ± 0.02
15	−1.25 ± 0.02	0.78 ± 0.01	−1.20 ± 0.02	0.76 ± 0.02
16	−1.24 ± 0.02	0.78 ± 0.01	−1.20 ± 0.03	0.76 ± 0.03
17	−1.24 ± 0.02	0.79 ± 0.01	−1.19 ± 0.02	0.78 ± 0.02
18	−1.24 ± 0.01	0.79 ± 0.01	−1.19 ± 0.02	0.78 ± 0.02
19	−1.23 ± 0.02	0.80 ± 0.01	−1.19 ± 0.02	0.79 ± 0.02
20	−1.23 ± 0.02	0.80 ± 0.02	−1.19 ± 0.02	0.80 ± 0.02

Each reported value represents the average of 30 independent simulations, with the corresponding standard deviation provided.

**Fig. 5:**
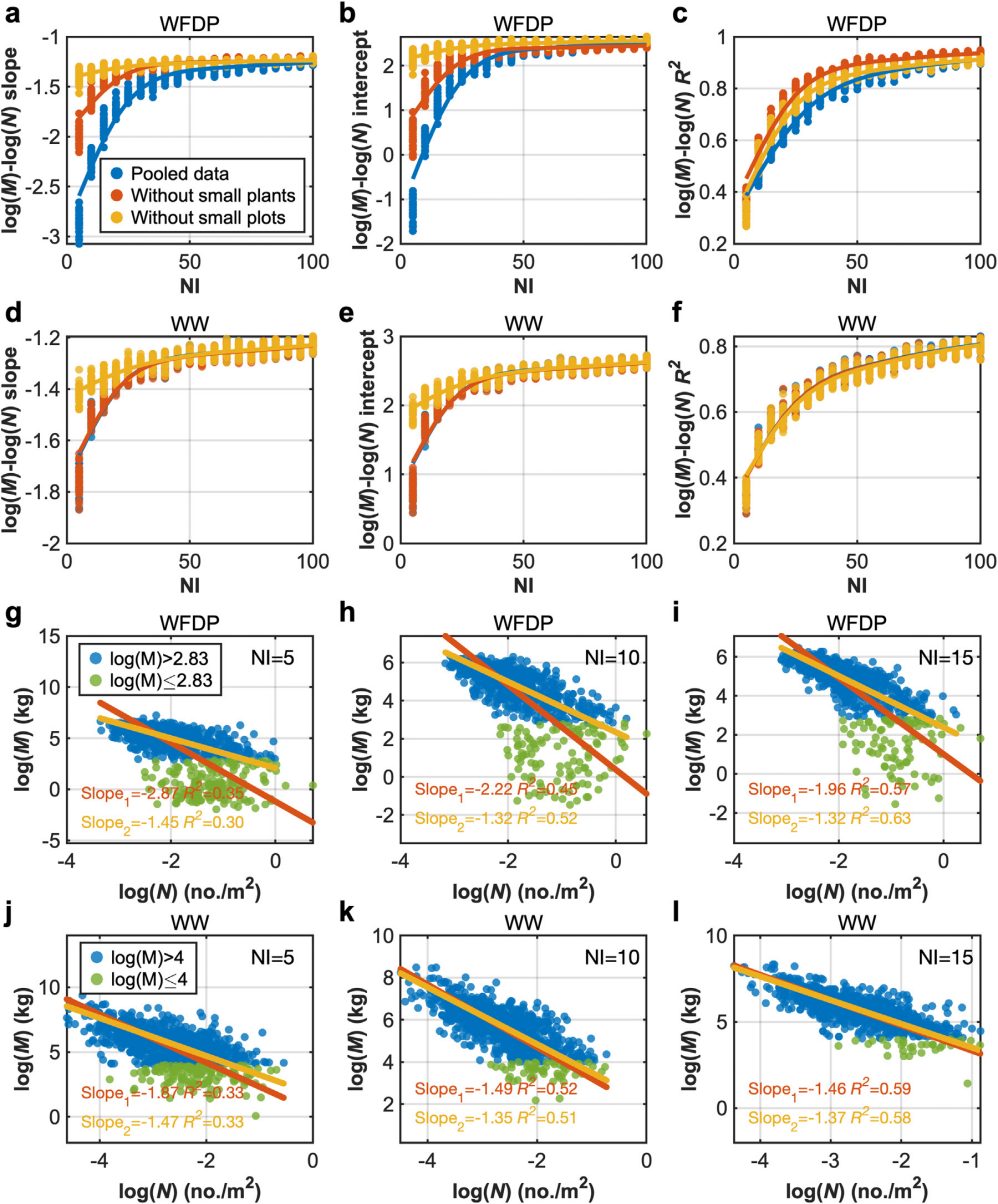
**Comparisons of the results obtained using the Voronoi diagram-based method with those from the other two scenarios, along with a demonstration of the impact of scenario 2**. The two scenarios are described as follows: (1) without small plants (conducting simulations using plant individuals with biomass > 1 kg); (2) without small plots (conducting simulations with all individuals and fitting plot data with a high value of log(average biomass)). (a-f) The variation in the slopes, intercepts, and corresponding *R*^2^ values of the log(average biomass)-log(density) (log*M*-log*N*) relationship across different NI values for the three scenarios, respectively. Each point represents the result of a single simulation, with the solid lines fitted using Generalized Additive Models. (g-i) Comparisons of the linear relationship between log(average biomass) (log*M*) and log(density) (log*N*) before and after excluding plot data with log*M* ≤ 2.83 at WFDP. (j-l) Comparisons of the linear relationship between log(average biomass) and log(density) before and after excluding plot data with log*M* ≤ 4 at WW. Blue points represent plot data with high log(average biomass) values, while green points correspond to plot data with low log(average biomass) values. The yellow lines represent the relationship obtained by fitting all plot data using Reduced Major Axis regression (RMA), while the red lines represent the fit using only the blue points. Each figure specifies the dataset and the number of individuals per plot (NI) used in the simulation, along with the RMA-fitted slope and the corresponding *R*^2^ value. All fitted results are significant at the *P* < 0.001 level.

Both the presence of small individuals and small plots contribute to variation in log*M*-log*N* slopes across NI, with small plots exerting a stronger influence. In both scenario 1 (performing simulation for plant individuals with biomass > 1 kg) and scenario 2 (performing simulation for all individuals and fitting plot data with high values of log(average biomass)), the variability in the slope and intercept of the log(average biomass)-log(density) relationship was reduced at WFDP (Fig. 5a–f). However, scenario 2 also significantly reduced this variability at WW, whereas scenario 1 had minimal impact on the variability at WW. The variability of the scaling exponent for the two scenarios is 0.038 and 0.0074 at WFDP, and 0.015 and 0.0044 at WW, respectively. In the first case, both scenarios show lower variability than the original value of 0.075 at WFDP. In the second case, scenario 2 exhibits a notably reduced variability compared to the original 0.016 at WW. Similarly, the variability of the intercept for the two scenarios is 0.11 and 0.019 at WFDP, and 0.12 and 0.029 at WW, respectively, compared to the original values of 0.18 at WFDP and 0.12 at WW. These variability measures were derived from data where NI ≤ 20 at WFDP and NI ≤ 15 at WW. While the slope and intercept for scenario 1 still show clear variation with NI, the changes in slope and intercept for scenario 2 are less pronounced (Fig. 5a–f). Both scenarios result in a slight increase in *R*^2^ values at WFDP, but the overall trend remains unchanged (Fig. 5a, d). As NI increases, fewer plot data are excluded, and conversely, excluding more plot data leads to a greater influence on the scaling exponent (Figs. 5g–l and S9, S10).

Biomass estimation is more accurate using Voronoi diagram-based spatial partitioning than using overall forest density and area alone. Based on the estimated log*M*-log*N* relationship under the NI = 5 simulation condition from the Voronoi diagram-based approach, we predicted the total biomass of the WFDP plot as 4.88 × 10^6^ kg This value is approximately 0.2% lower than the biomass estimated by the “allodb” package (4.89 × 10^6^ kg). Similarly, for the WW plot, we predicted the total biomass as 4.49 × 10^6^ kg under the NI = 15 simulation condition, which is about 1% higher than the “allodb” estimate (4.45 × 10^6^ kg). Using the same log*M*-log*N* scaling equation, when applying the total area and density of the forest plot, the predicted biomass for WFDP is 3.97 × 10^6^ kg, approximately 19% lower than the “allodb” estimate. Similarly, the predicted biomass for WW is 3.72 × 10^6^ kg, about 16% lower than the “allodb” estimate.

## 4 Discussion

### 4.1 Advantages of the Voronoi diagram-based approach

The Voronoi diagram-based method enhances density estimation by adapting to spatial heterogeneity, outperforming quadrat-based sampling. For plants exhibiting a regular grid arrangement, quadrat-based methods can provide accurate density estimates (Fig. 1a). However, in natural systems characterized by irregular patterns, quadrat-based sampling methods often encounter difficulties in determining the boundaries and locations of quadrats, which may require substantial expertise and experience (Fig. 1a). This challenge could explain the use of multiple quadrat sizes in previous field surveys [1,3,7]. In contrast, the Voronoi diagram-based method offers a more objective and precise density estimation, capable of handling both regular grid and naturally irregular patterns, thereby overcoming the limitations of quadrat-based methods (Fig. 1b). Both the Voronoi diagram-based and quadrat-based methods showed similar trends in log*M*, log*N*, and log*M*-log*N* relationship across NI or Avg.QSL (Figs. 3 and S11), but the Voronoi diagram-based method yielded higher *R*^2^ values, especially at smaller NI or Avg.QSL (Fig. 4), while the lower *R*^2^ under the quadrat-based method cannot be explained by the variation in plant density or biomass (Fig. S12). The Voronoi diagram-based method achieves a higher model fit (*R*^2^) than the quadrat-based method, offering a more reliable understanding of mass-density dynamics and deeper insights into population behavior, particularly within self-thinning contexts. This approach effectively captures irregular plant communities, offering a novel perspective for analyzing complex ecosystems like forests and providing a more precise tool for ecological research. Furthermore, it facilitates future ecological studies and practical applications in ecosystem monitoring and management.

In forest research, the individual plant is the fundamental sampling unit. However, traditional fixed-area plots have often failed to ensure a consistent number of individuals, resulting in variable data quality, especially in ecosystems with heterogeneous spatial distributions (Fig. 1a). To address this, ecologists often use quadrats of varying sizes in regions with large density fluctuations, such as deserts and grasslands [1,7]. Yet, even within a single ecosystem, spatial aggregation frequently undermines the reliability of fixed-shape plots. To overcome these limitations, we adopted a Voronoi diagram-based sampling approach that reversed the conventional logic of traditional methods: instead of fixing area and counting individuals, we fixed NI and determined the area they occupy using Voronoi tessellation. Each individual was enclosed within a polygon defined by perpendicular bisectors between it and neighboring individuals. This individual-based method adapts the sampling area to local density, yielding larger polygons in sparse regions and smaller ones in dense regions, thereby enhancing sampling flexibility and accuracy, particularly in structurally complex environments such as mountainous forests.

This spatial partitioning approach offers two key advantages. First, it circumvents the positional bias inherent in quadrat-based methods, where arbitrary plot placement can result in substantial variation in individual counts, particularly when individuals are clustered near plot boundaries (Fig. 1a). Second, because Voronoi cells capture local spatial structure, the resulting density estimates are both high-resolution and ecologically meaningful. To further enhance accuracy, we average the areas of multiple adjacent Voronoi cells to account for interactions beyond immediate neighbors and to reduce stochastic variation caused by random spatial distribution. A notable limitation emerged in regions of extremely high individual density: minor inaccuracies in area estimation can lead to disproportionately large fluctuations in density values due to the inverse area-density relationship. Thus, although the Voronoi diagram-based method enhanced sampling precision and adaptability, its application in high-density contexts requires careful parameter tuning and consideration of fine-scale spatial heterogeneity.

### 4.2 Key impact factors influencing the scaling exponent

Excluding small individuals and plots stabilizes the mass-density scaling exponent, enabling more reliable estimation with lower sampling effort. In biomass-density studies, obtaining a stable log*M*-log*N* relationship remains a central goal during field sampling. Our study revealed a previously undocumented pattern: the slope of the log*M*-log*N* relationship varied significantly with NI, particularly at low NI levels (Fig. 4). This variability raises concerns regarding the reliability of mass-density estimates derived from limited samples and warrants further investigation into its underlying causes. As shown in Fig. 3, fluctuations in slope are often associated with the presence of small individuals or plots with low log*M* values [28]. Such individuals are typically young, experience high turnover rates, and often lack evidence of interaction or self-thinning. They are also more susceptible to measurement errors in both biomass and abundance estimations. In large forest plots, prolonged survey periods further exacerbate these issues, as small individuals may grow, die, or recruit during the census interval. In contrast, larger individuals tend to be spatially stable and temporally consistent, thereby yielding more consistent and reliable estimates. Accordingly, the selective exclusion of small individuals or low-biomass plots may enhance the stability and accuracy of mass-density relationships.

To examine whether changes in the scaling exponent with NI are driven by small plants or plots, we introduced scenario 1 (conducting simulations for plant individuals with biomass > 1 kg) and scenario 2 (conducting simulations for all individuals and fitting plot data with a high value of log(average biomass)). Scenario 2 markedly reduced the variability of the scaling exponent across NI at both WFDP and WW compared to scenario 1 (Fig. 5a–f), suggesting that small plots are a key factor influencing the scaling exponent. The fitting results for the second scenario (Figs. 5g–l and S9, S10) indicate an obvious increase in the slope after excluding small plot data, with the impact on the slope becoming more pronounced as more outliers were excluded.

Size inequality can reduce the scaling exponent, as a faster decline in density than size increase among smaller individuals prompts the exclusion of these individuals to explore changes in the exponent [28]. In the first scenario, size inequality was reduced, leading to a notable decrease in the plot Gini coefficient [44], the coefficient of variation for plant density and average biomass at WFDP (Fig. S13). In comparison, scenario 2 revealed that small plots were the primary contributors to the substantial variation in the scaling exponent with changes in NI. Under lower NI conditions, a higher frequency of small plot data was observed compared to higher NI conditions (Figs. 3c–d and S9, S10). This pattern explains the more substantial variation in scaling exponent at lower NI values and its attenuation as NI values increase (Fig. 4). Under lower NI conditions when more small plants are sampled, the calculated log (average biomass) values are likely to be small. As NI increases, although more small plants remain within the sample, the influence of larger individuals increasingly dominates the average effect, resulting in higher log (average biomass) values that prevent the plot from being classified as a small plot. This sampling phenomenon is likely attributed to the formation of forest gaps, which create localized areas with higher densities of smaller individuals due to increased light availability and reduced competition. Consequently, sampling with smaller NI may yield lower average biomass values, leading to deviation from the overall distribution pattern.

When conducting field sampling using the Voronoi diagram-based method to investigate the scaling relationship between plant average biomass and density, it is generally desirable for the scaling exponent to show minimum sensitivity to changes in NI, thereby ensuring a reliable and stable mass-density relationship. However, the scaling exponent showed a significant increasing trend with smaller NI values (Fig. 4), while higher NI entailed significantly greater sampling cost. When small plots with log (average biomass) were excluded during fittings in scenario 2, the variability across NI decreased from 0.075 to 0.0074 at WFDP, and 0.016 to 0.0044 at WW, respectively (Fig. 5a–f). This scenario allows for reliable sampling at lower NI levels while maintaining a stable scaling relationship. This finding significantly enhances the reliability of data analysis and sampling efficiency, enabling the derivation of stable mass-density relationships with a lower investigation scale, thereby reducing fieldwork costs. Moreover, the method holds considerable potential for informing ecosystem management, advancing both theoretical understanding and applied ecological research.

The mass-density relationship encapsulates interaction-driven ecological dynamics, wherein competition for limited resources typically leads to lower individual biomass at higher densities. While most forest surveys and mass-density studies focus on large trees, small individuals particularly seedlings, are often excluded due to their limited competitive ability and resource acquisition [36]. In this study, we evaluated the mass-density relationship both with and without small individuals. In forest ecosystems, long census intervals, high variability, and greater measurement uncertainty make biomass estimation for small individuals particularly challenging. Including them can disproportionately reduce mean biomass and inflating density, thereby distorting the scaling relationship. Moreover, they often contribute little to the interaction-driven processes that underpin this pattern. Accordingly, excluding small individuals can enhance both the accuracy and stability of mass-density estimates. Similarly, smaller plots tend to overrepresent small individuals, introducing comparable bias. Therefore, omitting both small individuals and small plots may provide a more reliable basis for empirical assessment of mass-density relationships.

### 4.3 Applications

The plant mass-density relationship serves as a pivotal framework for forest biomass prediction and density management [21,45]. Based on the scaling relationship derived from random sampling simulations, we can predict the total biomass of the entire forest plot. Biomass predictions based on Voronoi diagram-derived area and density closely match “allodb” estimates [37], with deviations within ±1%. These simulations were based on biomass estimates from “allodb”, which were used to derive the allometric log*M*-log*N* relationship. For both WFDP and WW, the biomass predictions obtained under these specific NI conditions showed the smallest deviations and were therefore adopted as the final biomass estimates. However, as the intercepts and slopes still exhibit variability across different NI values (Fig. 5a–f), using intercepts and slopes from alternative NI simulation conditions yielded considerably larger biases in the total biomass prediction. Applying the same log*M*-log*N* equation to total plot values resulted in biomass estimates 16%−19% lower than “allodb” estimates. This demonstrates that the biomass estimation based on spatial partitioning using Voronoi diagrams is significantly more accurate than the method relying solely on overall forest density and area. Remarkably, the differences between our biomass predictions and those derived using the “allodb” method are minimal. This benchmark not only validates the reliability and precision of our approach but also highlights its substantial potential for practical applications. Unlike conventional prediction method that uses overall density and area, our approach captures individual-level heterogeneity, providing a more realistic reflection of forest ecosystem dynamics. Consequently, this approach provides a robust, scalable tool for accurate biomass prediction in forest ecosystems.

The Voronoi diagram-based method can be effectively applied to guide field experiments and assess plant density in agricultural settings. Traditional field density experiments often rely on density gradients established across multiple plots, which typically result in a narrow range of density variation, increased labor demands, reduced flexibility, and a failure to account for spatial heterogeneity in farmland. In contrast, by applying our Voronoi diagram-based approach, plant densities can be estimated within a single large experimental plot using spatial coordinates of randomly sown individuals. This enables more diverse and flexible density distributions, enhancing the analysis of mass-density relationships compared to conventional gradient-based sowing methods.

This method also holds promise for studies of plant interactions, as its core principle involves partitioning space based on the spatial relationships between neighboring individuals. By integrating key competitive traits (e.g., height, stem diameter, crown size) as weighting factors within the spatial partitioning, this method can be extended to better reflect asymmetric competition. As demonstrated in this study, merging multiple Voronoi polygons allows competition to be assessed over broader spatial scales, supporting the investigation of inter-individual interactions. Furthermore, given that density estimation is performed at the individual level, this method is particularly well-suited for exploring spatial heterogeneity in plant communities.

### 4.4 Limitations of the Voronoi diagram-based approach

Our study applied the Voronoi diagram-based method within two forest plots (WFDP and WW) to evaluate its effectiveness; however, future investigations are essential to test its generality across a broader spectrum of ecosystem types. Because this method requires precise spatial coordinates of individual plants to partition space, it is particularly well-suited for ecosystems such as drylands, shrublands, and croplands, where individual locations are readily distinguishable. In contrast, in ecosystems like grasslands or dense shrublands, where individuals are not spatially distinct, determining their positions can be challenging, thereby limiting the method’s applicability. To extend its application, subsequent research could explore experimental dryland or agricultural plots with known spatial configurations or integrate high-resolution remote sensing data [46]. Individual-based remote sensing approaches offer the potential to accurately map plant spatial distributions, rendering them particularly suitable for scaling up the application of this method.

In our study, plant biomass in the WFDP and WW plots was estimated using allometric equations rather than through direct measurements [37], which are more common in controlled agricultural field experiments. This may have influenced the observed mass-density relationships. While biomass estimation inherently contains uncertainty, such errors may be acceptable within specific ecological contexts. Despite these limitations, the mass-density relationship derived from Voronoi diagram-based sampling provides a valid framework for biomass estimation. Its application—inferring total biomass from density and area estimates remains methodologically sound. However, further validation against biomass estimates derived from remote sensing approaches remains a critical next step.

The choice between Voronoi diagram-based and quadrat-based methods entails a scale-dependent trade-off. While the new method offers higher accuracy at finer spatial scales, its relative advantage diminishes with increasing spatial scale, making quadrat-based sampling more practical and efficient for broader-scale applications (Figs. 4 and S3). Importantly, implementing the Voronoi diagram-based method in field surveys does not require spatial coordinates for all individuals. Instead, by recording the positions of focal individuals and their immediate neighbors, irregular plot boundaries can be constructed using Voronoi tessellation (as shown in Fig. 1b). This allows effective spatial partitioning based on partial spatial data, enhancing its applicability in real-world settings where comprehensive individual mapping is impracticable.

## 5 Conclusion

Our proposed Voronoi diagram-based sampling approach outperformed the quadrat-based method in terms of accuracy and efficiency. This approach proves to be a highly effective tool for analyzing the mass-density relationship in complex ecosystems and holds significant potential for broad ecological applications. Moreover, as NI increased, the scaling exponent initially increased significantly before stabilizing, primarily due to the presence of more small plots with smaller log (average biomass) values at low NI conditions. This finding improves both the reliability of data analysis and sampling efficiency, as excluding small plots (in scenario 2) enables the achievement of a more stable scaling relationship even with a smaller NI, thereby lowering the overall survey effort. Furthermore, spatial partitioning via Voronoi diagrams enables more accurate predictions of total biomass. Beyond biomass estimation, this method offers valuable utility in the design and guidance of density-controlled experiments and modeling of plant interactions. Looking forward, we aim to expand this research by incorporating data from additional forest sites and diverse ecosystems to further validate the applicability of this method across different ecological contexts.

## Declaration of generative AI and AI-assisted technologies in the writing process

During the preparation of this work the authors used ChatGPT in order to optimize and refine some grammar. After using this tool, the authors reviewed and edited the content as needed and take full responsibility for the content of the publication.

## CRediT authorship contribution statement

**Liang Zhang:** Writing – review & editing, Writing – original draft, Methodology, Formal analysis. **Shubin Xie:** Writing – original draft, Methodology. **Renfei Chen:** Writing – original draft, Formal analysis. **Jinzhi Ran:** Validation, Supervision. **Jianming Deng:** Project administration, Funding acquisition.

## Declaration of competing interest

The authors declare that they have no conflicts of interest in this work.

## Appendix Supplementary materials

Image, application 1
