# Supplementary material for: A novel approach for sampling plant mass-density relationships
Source: Fundam Res. 2025 Jun 29;6(4):2343–54. doi: 10.1016/j.fmre.2025.06.009 (PMC13424157; doi:10.1016/j.fmre.2025.06.009)
Supplement: Supplementary file 1 [file mmc1.docx]

## Appendix A


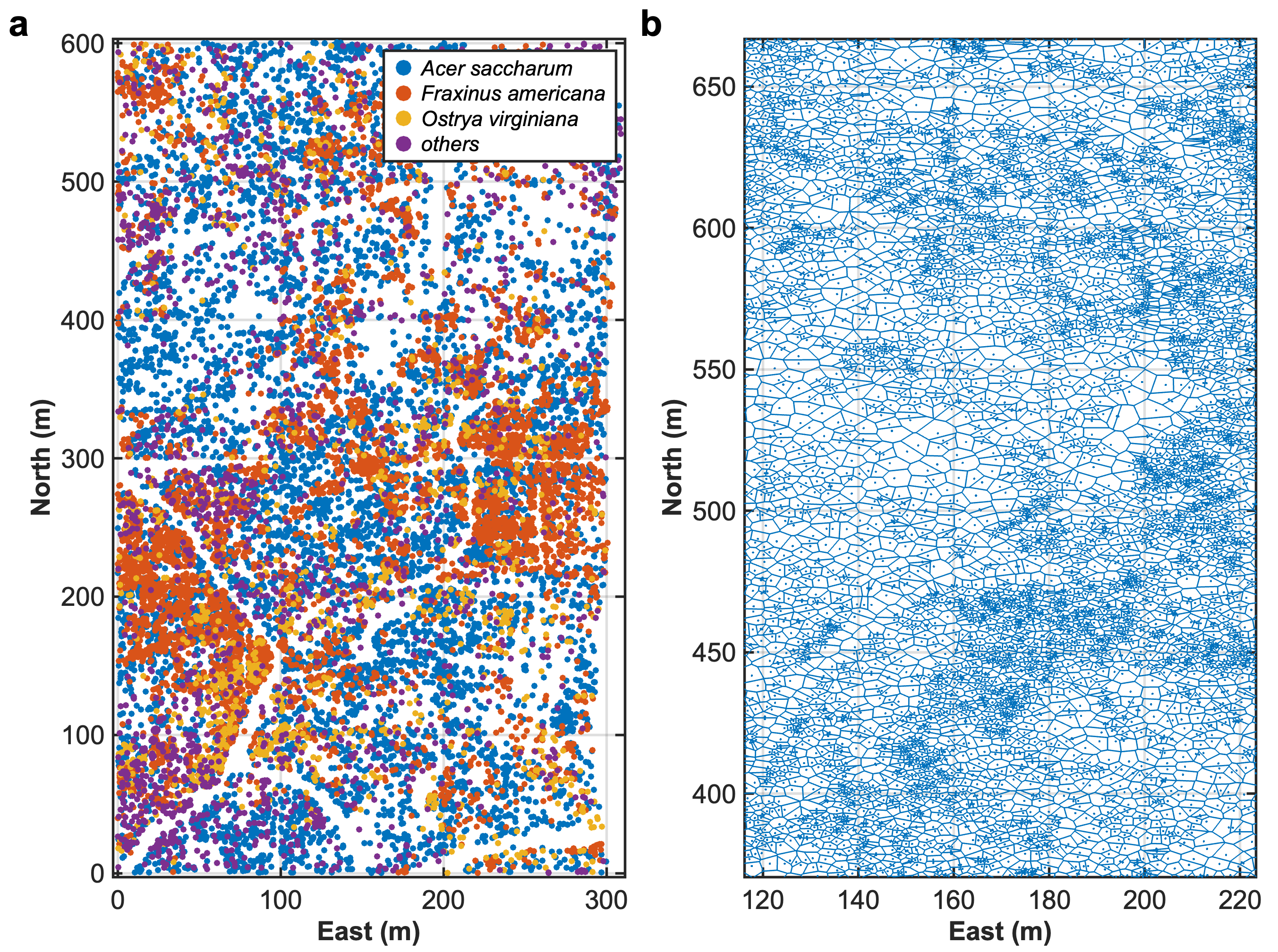


**Fig. S1.** S**patial distribution, and Voronoi diagram for part Wytham Woods (WW).** (a) Spatial distribution of the three most abundant species in WW, represented by points in three different colors. (b) Voronoi diagram partitioning for each plant in a part of WW.


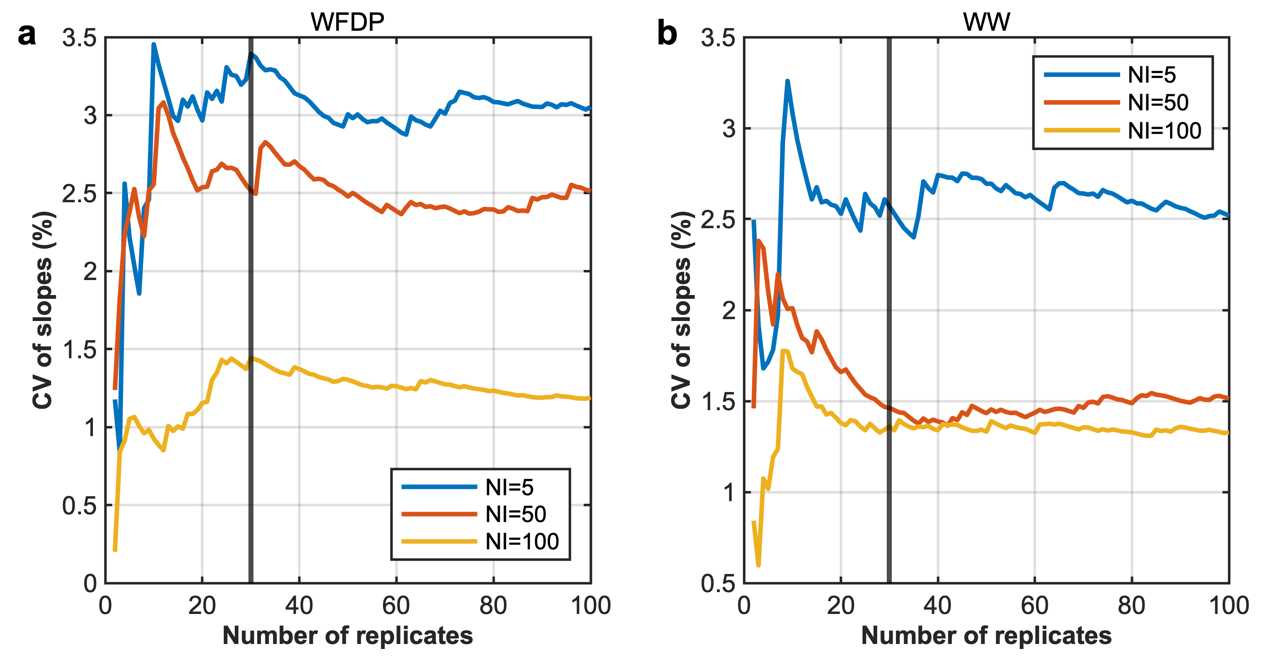


**Fig. S2 The coefficient of variation (CV) of the log(*M*)-log(*N*) slopes under the Voronoi diagram-based method is shown as a function of the number of replicates.** The blue, red, and yellow curves represent the results under the sampling conditions of NI = 5, 50, and 100, respectively. The vertical black line indicates the replicate number of 30.


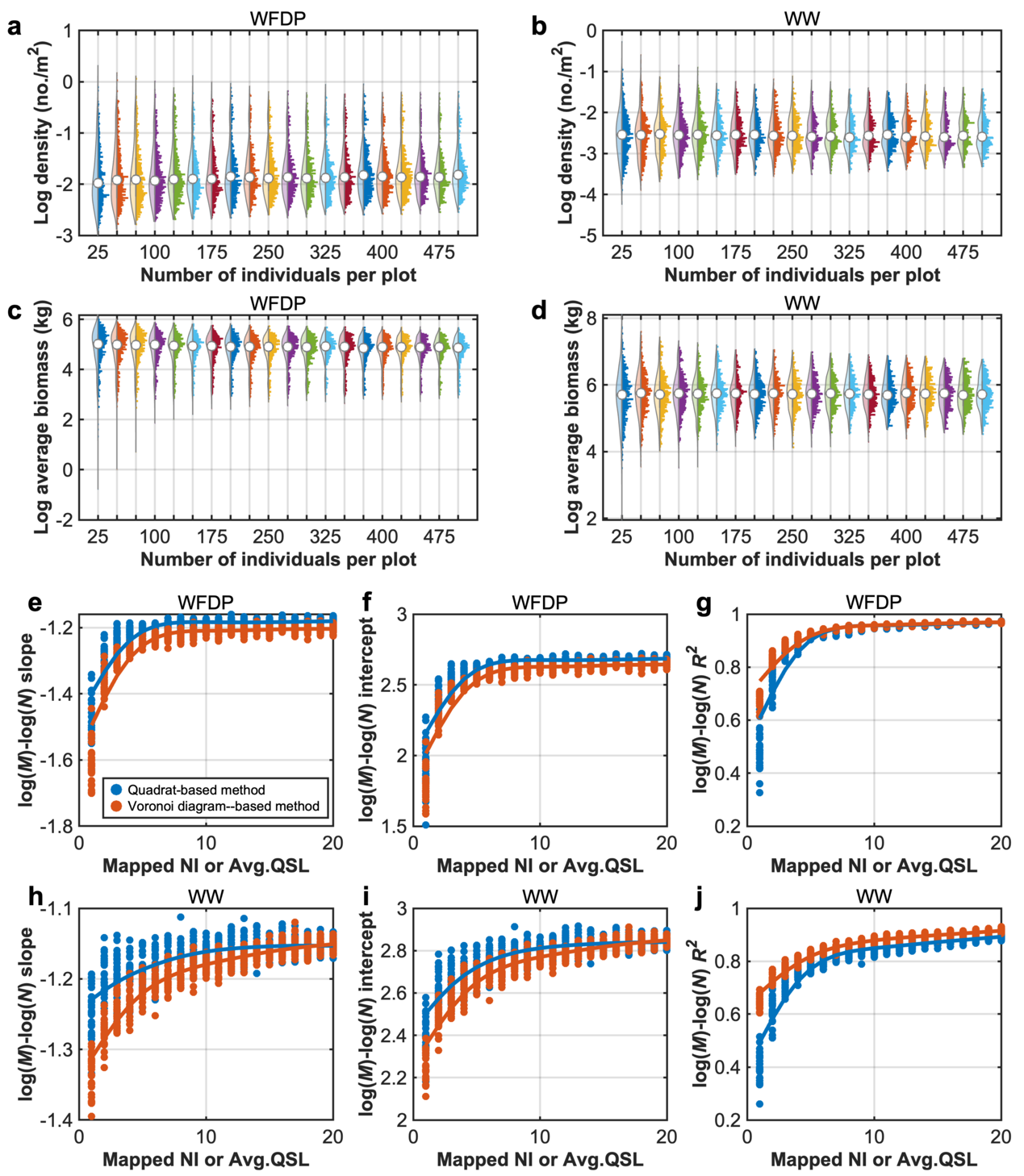


**Fig. S3 Distribution of simulated data using the Voronoi diagram-based sampling method (a–f), and comparisons of simulation results between the Voronoi diagram-based and quadrat-based methods (e–j).** The distributions of log(*N*) (a, b) and log(*M*) (c, d) were obtained from simulations under different NI values using the Voronoi diagram-based method, illustrated using both probability density functions and histograms. Comparisons of the slopes (a, d), intercepts (b, e), and corresponding *R*^2^ values (c, f) of the log(*M*)-log(*N*) relationship fitted using RMA for the Voronoi diagram-based method and the quadrat-based method are illustrated. Red points represent simulation results using the Voronoi diagram-based method, while blue points represent results from the traditional method. Each point corresponds to a single simulation, and the solid lines represent the trend fitted using GAMs. All RMA-fitted results are significant at the *P* < 0.001 level.


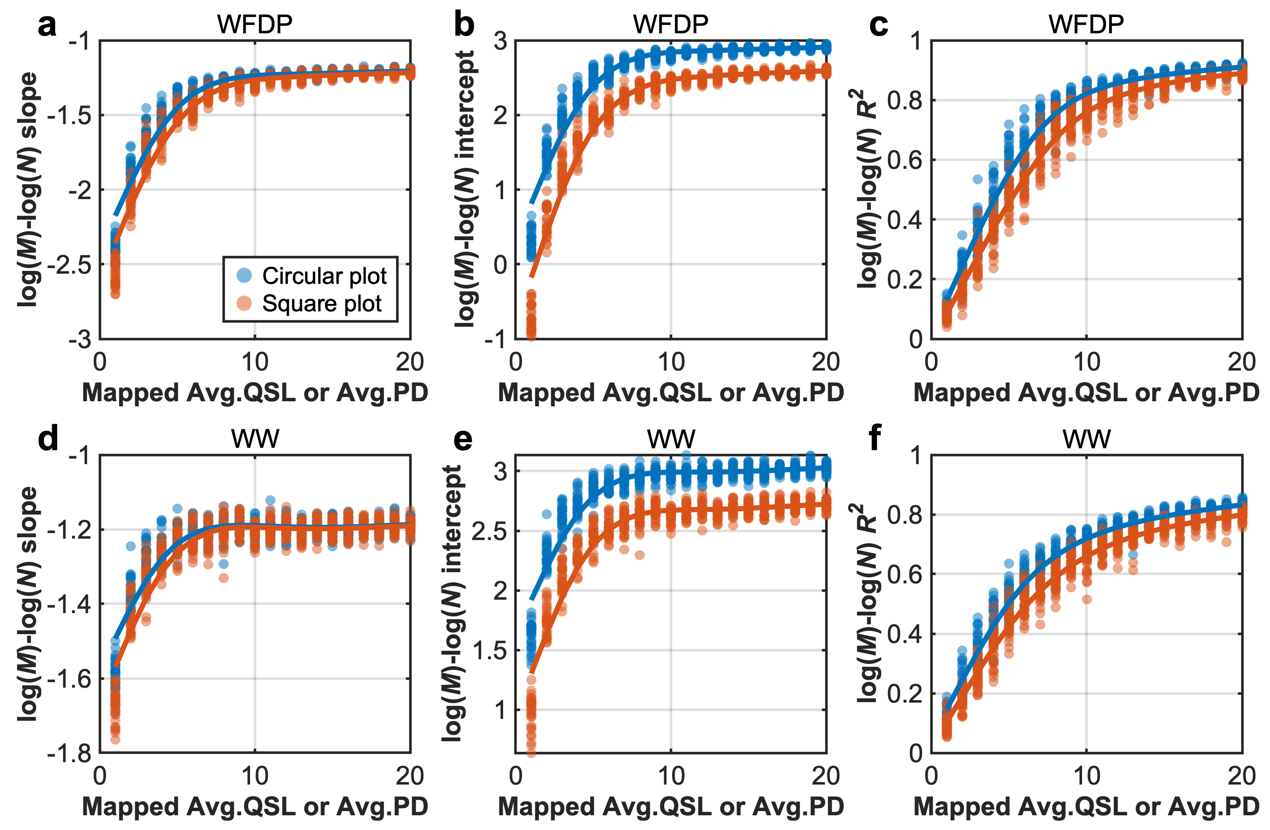


**Fig. S4.** **Comparisons of simulation results between different plot settings using the quadrat-based method.** Comparisons of the slopes (a, d), intercepts (b, e), and corresponding *R*^2^ values (c, f) of the log(*M*)-log(*N*) relationship fitted using the Reduced Major Axis regression for the quadrat-based method with different plot shapes are illustrated. Red points indicate the simulation results obtained using square plots, while blue points represent those based on circular plots. Each point corresponds to a single simulation, and the solid lines represent the trend fitted using Generalized Additive Models. All RMA-fitted results are significant at the *P* < 0.001 level.


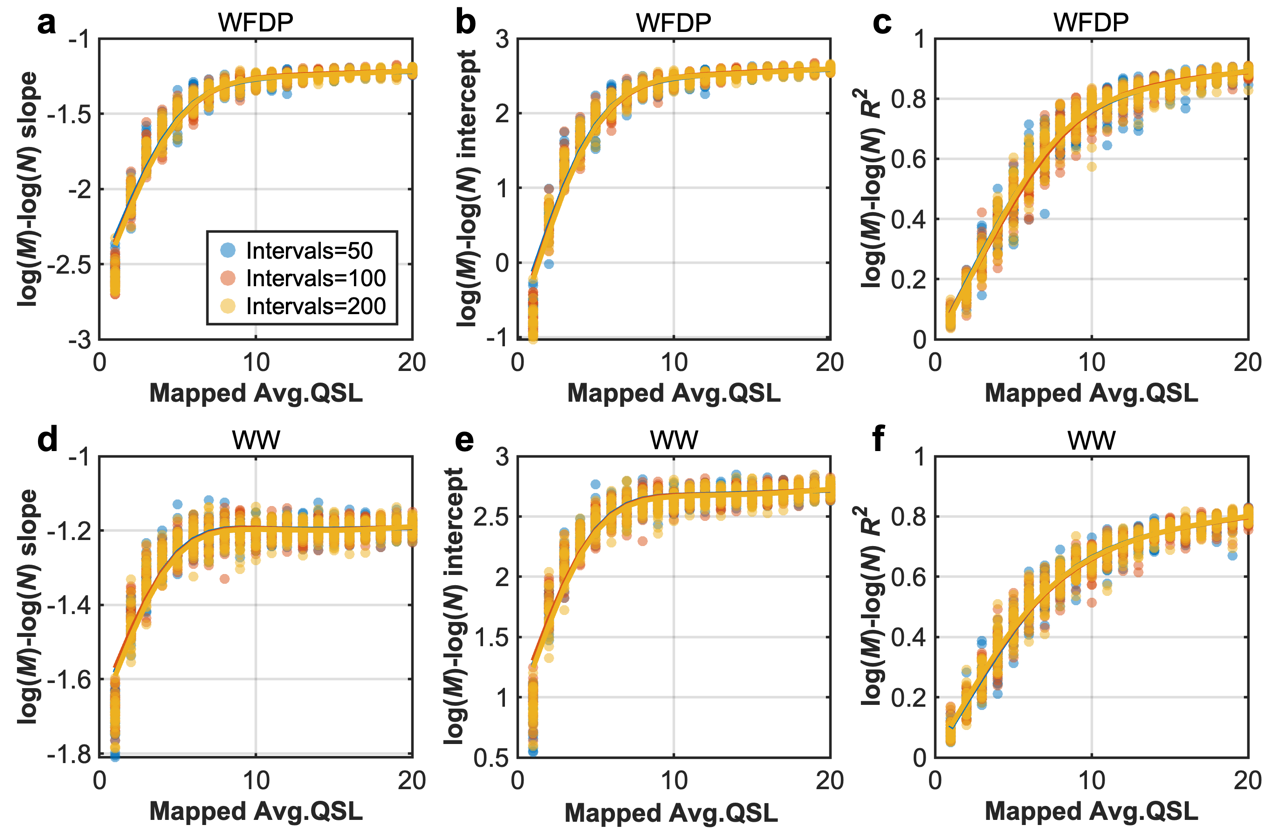


**Fig. S5.** **Comparisons of simulation results between different interval settings using the quadrat-based method.** Comparisons of the slopes (a, d), intercepts (b, e), and corresponding *R*^2^ values (c, f) of the log(*M*)-log(*N*) relationship fitted using the Reduced Major Axis regression for the quadrat-based method with different intervals are illustrated. Red points represent simulation results obtained with 50 histogram intervals, blue points correspond to results with 100 intervals, and yellow points indicate results using 200 intervals. Each point corresponds to a single simulation, and the solid lines represent the trend fitted using Generalized Additive Models. All RMA-fitted results are significant at the *P* < 0.001 level.


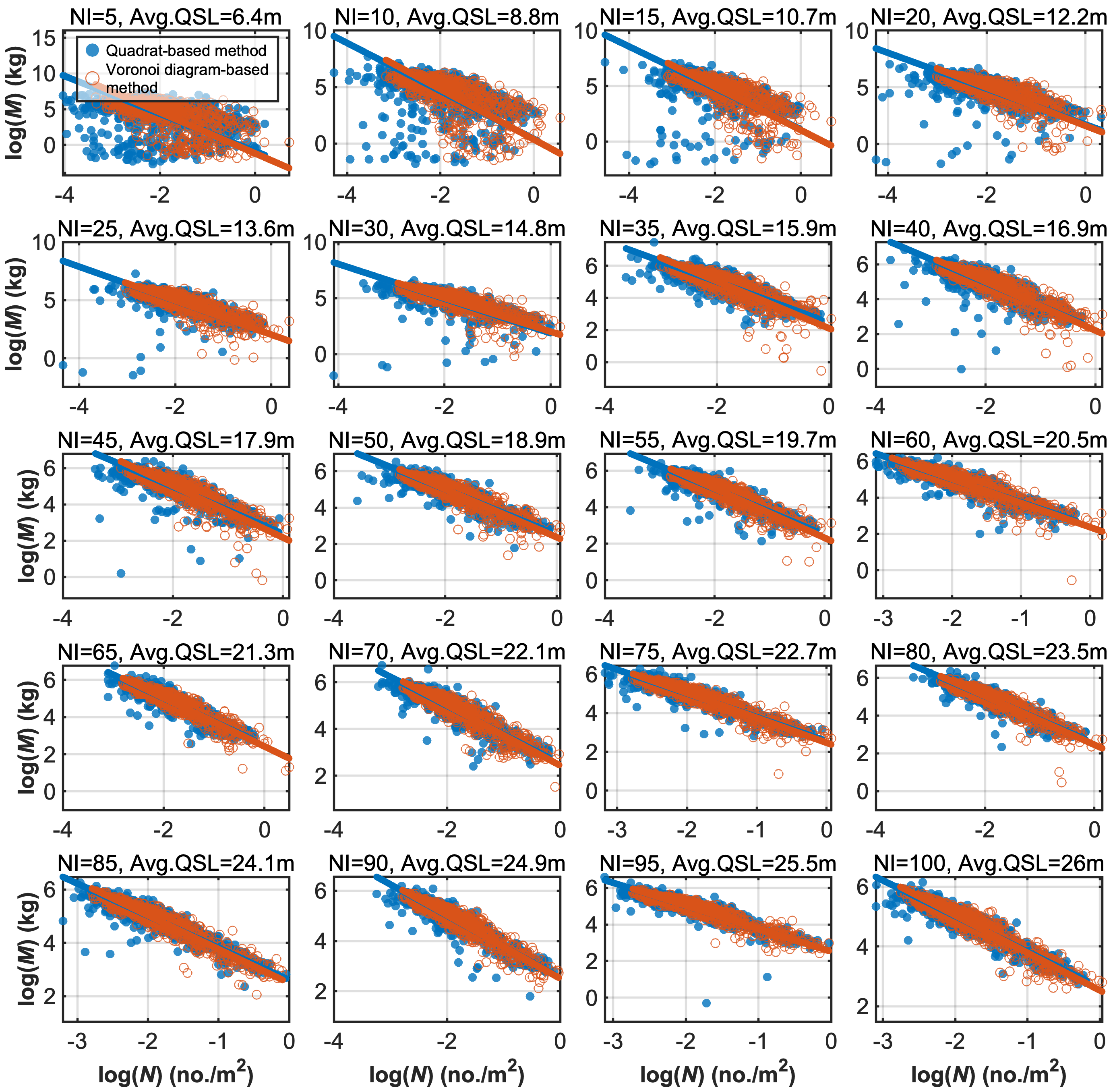


**Fig. S6.** **The linear relationship between log(average biomass) and log(density) under both the Voronoi diagram-based method and the quadrat-based method at WFDP.** Statistical regressions were performed using the Reduced Major Axis regression (RMA) method. Points represent the log(average biomass) and log(density) data, while the solid lines indicate the RMA-fitted lines. The plot title specifies the number of individuals (NI) used in the simulation (using the Voronoi diagram-based method) and the average quadrat side length (Avg.QSL) of the simulation (using the quadrat-based method). All fitted results are statistically significant at the *P* < 0.001 level.


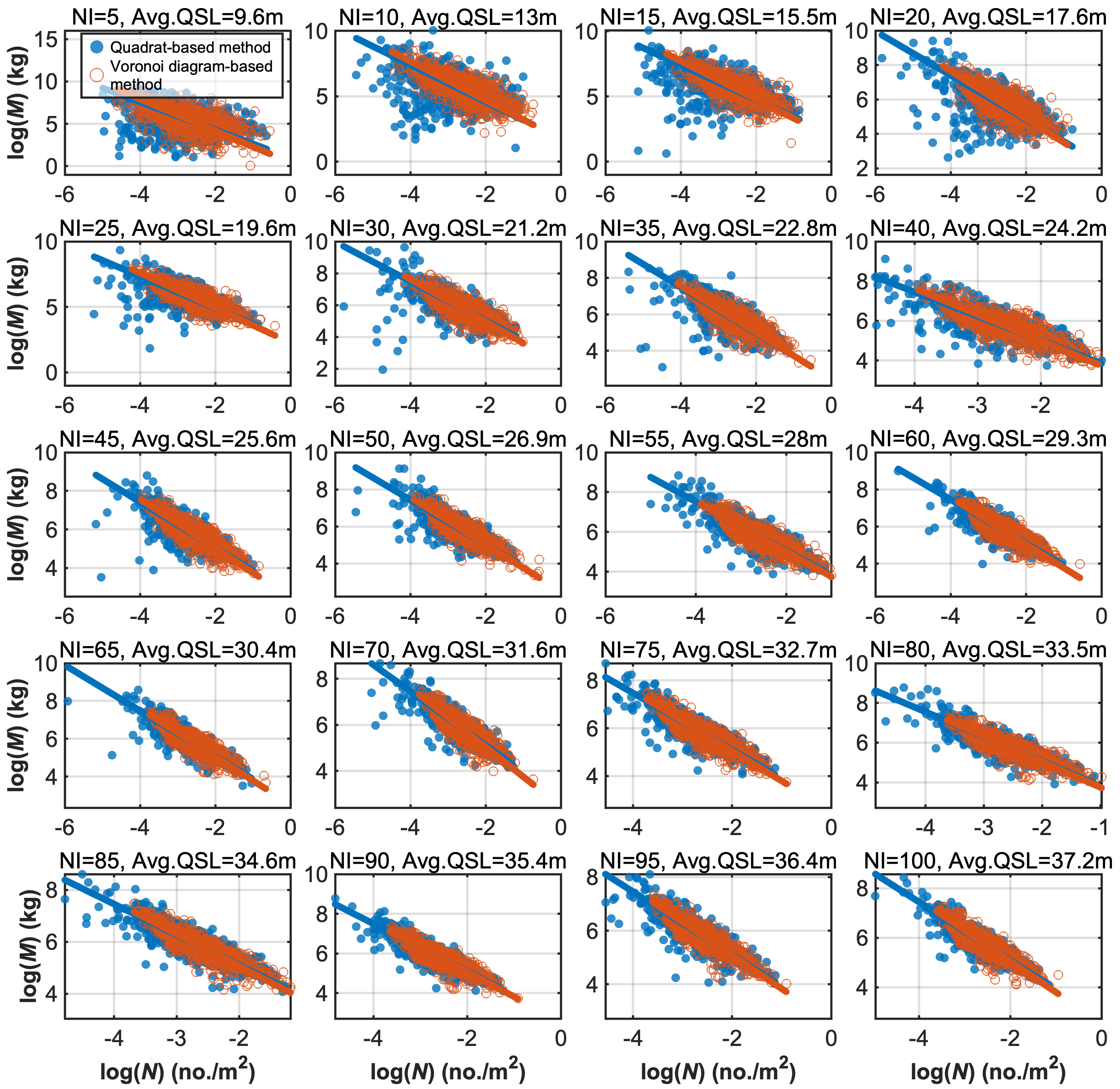


**Fig. S7.** **The linear relationship between log(average biomass) and log(density) under both the Voronoi diagram-based method and the quadrat-based method at WW.** Statistical regressions were performed using the Reduced Major Axis regression (RMA) method. Points represent the log(average biomass) and log(density) data, while the solid lines indicate the RMA-fitted lines. The plot title specifies the number of individuals (NI) used in the simulation (using the Voronoi diagram-based method) and the average quadrat side length (Avg.QSL) of the simulation (using the quadrat-based method). All fitted results are statistically significant at the *P* < 0.001 level.


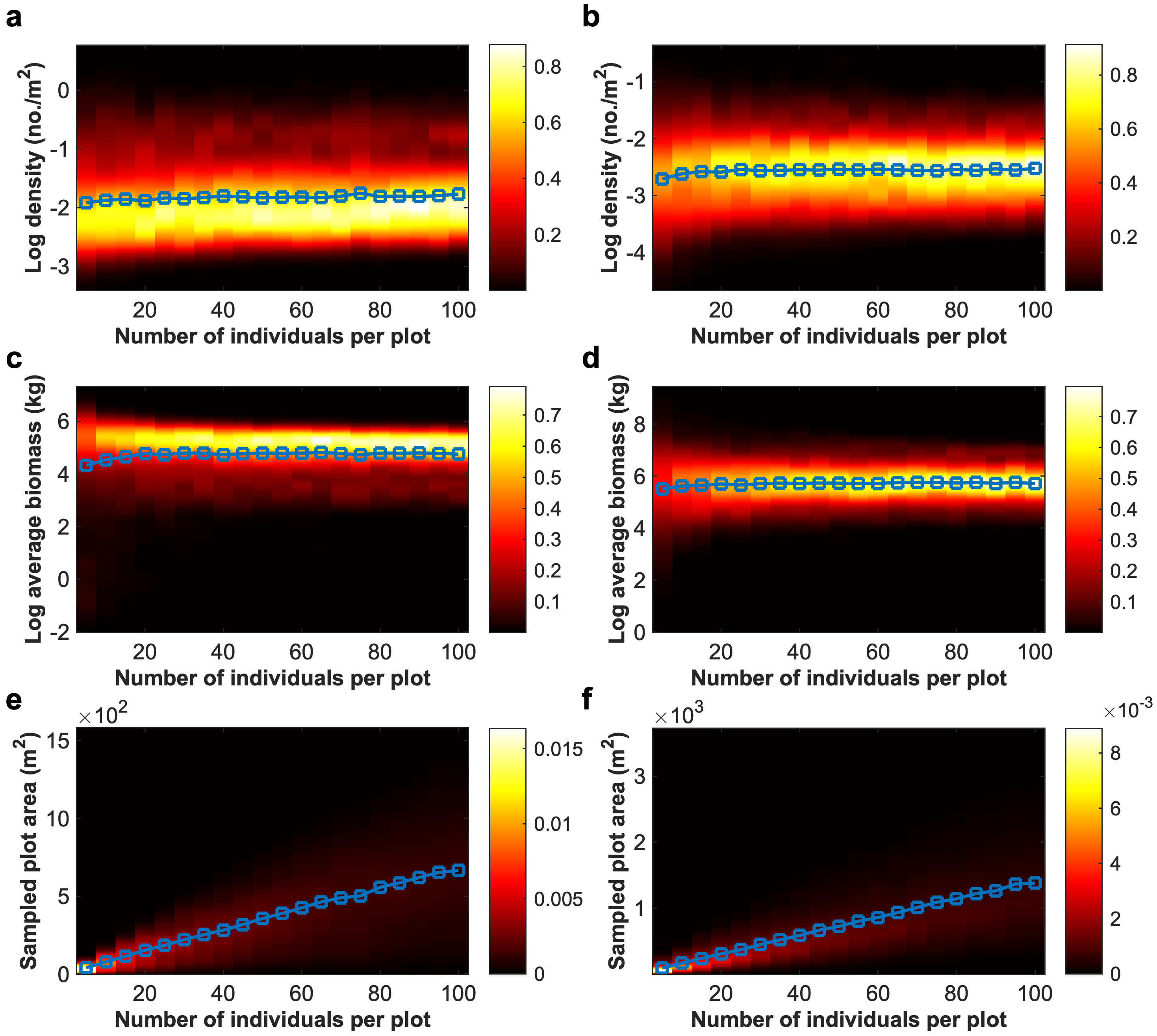


**Fig. S8.** **Simulation results of the Voronoi diagram-based sampling method.** (a, b) The distributions of log(density) were obtained from simulations under different NI (number of individuals per plot) values using the Voronoi diagram-based method, illustrated using heatmaps of probability density functions. (c, d) The distributions of log(average biomass) obtained using the Voronoi diagram-based method. (e, f) The distributions of the sampled plot area generated through the Voronoi diagram-based method.


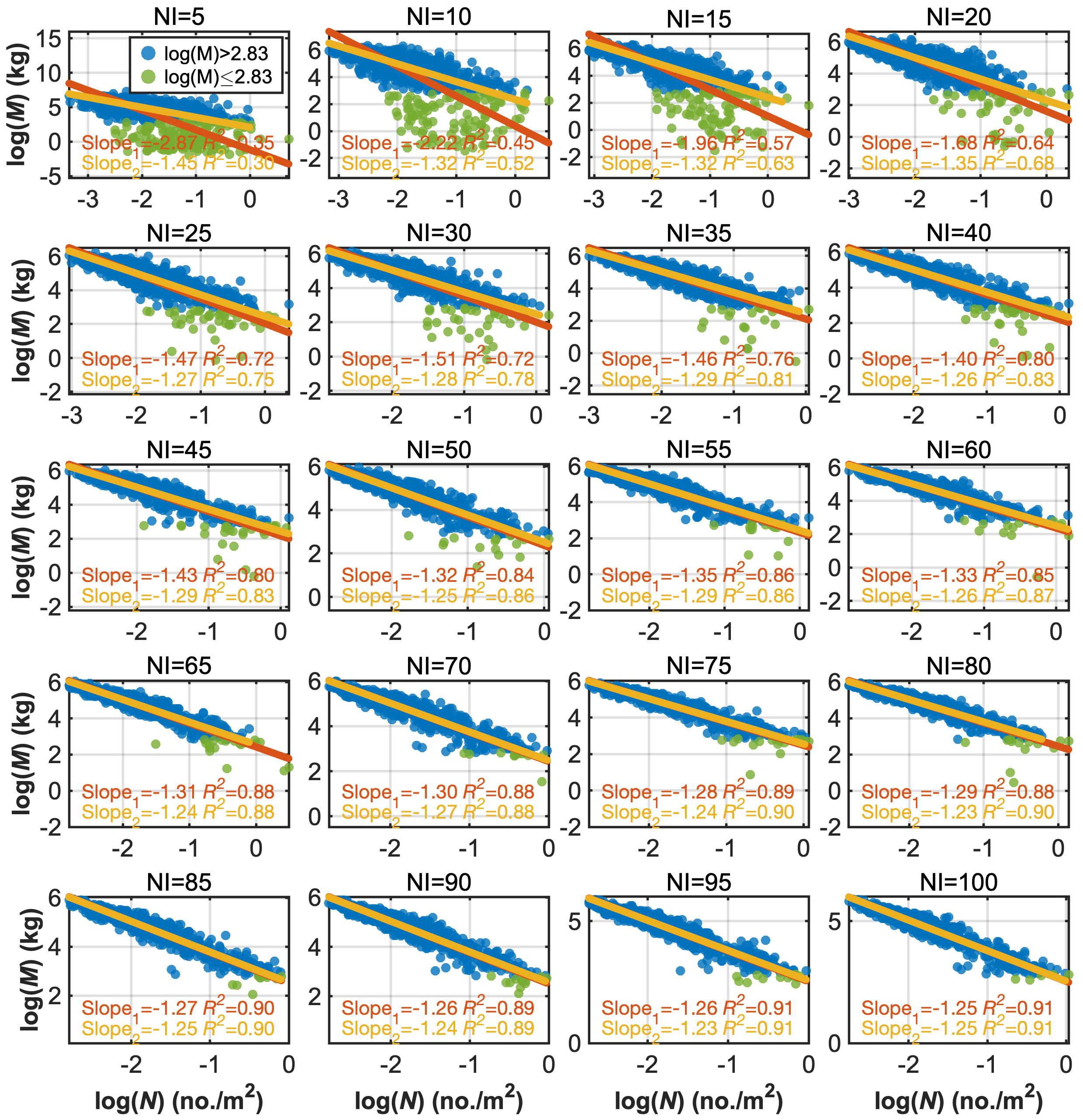


**Fig. S9.** **Comparison of the linear relationship between log(average biomass) and log(density) before and after excluding plot data with log(average biomass) ≤ 2.83 at WFDP.** Blue and green points represent plot data with log(average biomass) > 2.83 and log(average biomass) ≤ 2.83, respectively. The yellow lines represent the relationship obtained by fitting all data using Reduced Major Axis regression, while the red line represents the fit using only the blue points. Each figure specifies the number of individuals per plot (NI) used in the simulation, along with the RMA-fitted slope and the corresponding *R^2^* value. All fitted results are significant at the *P* < 0.001 level.


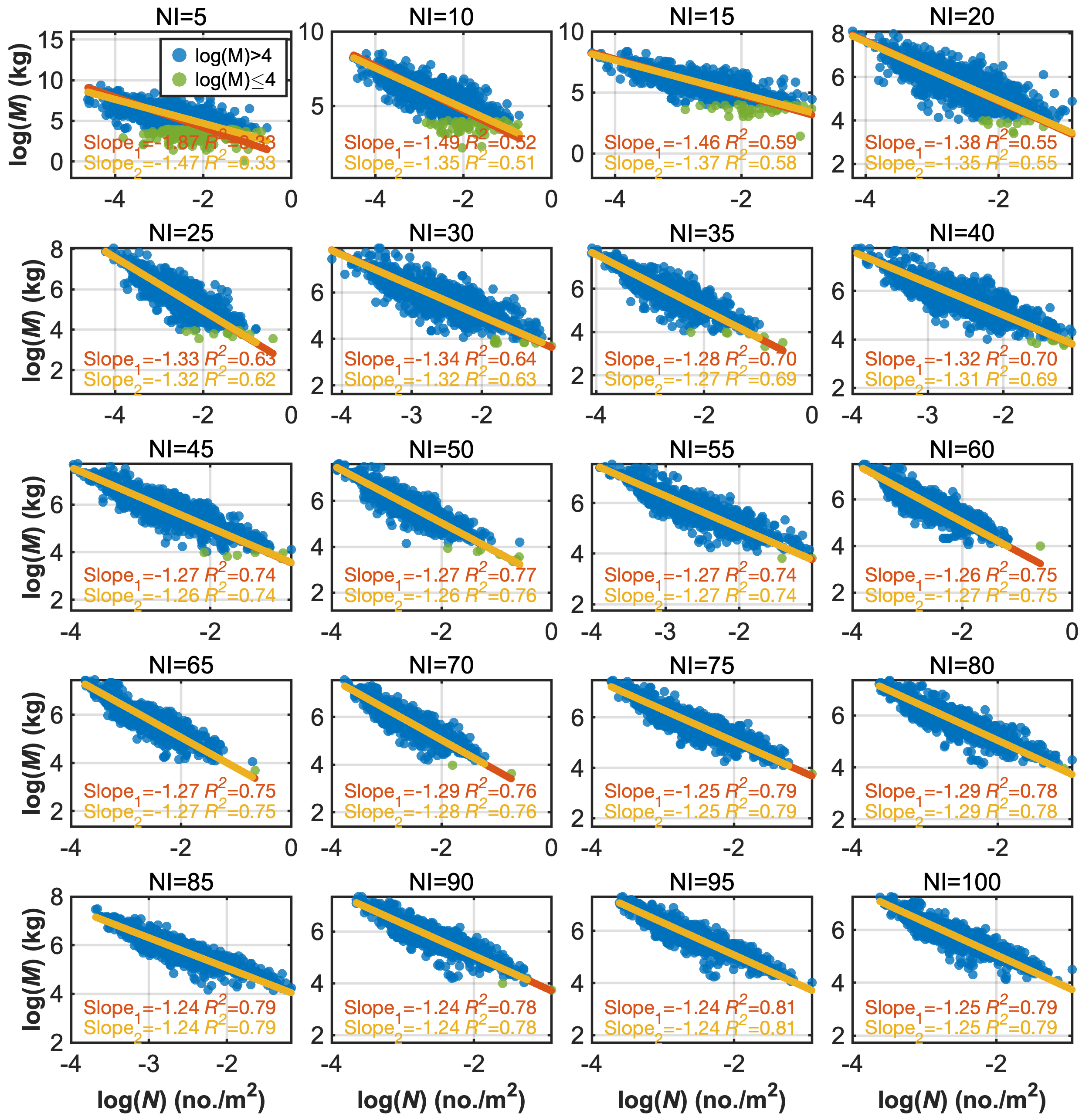


**Fig. S10.** **Comparison of the linear relationship between log(average biomass) and log(density) before and after excluding plot data with log(average biomass) ≤ 4 at WW.** Blue and green points represent plot data with log(average biomass) > 4 and log(average biomass) ≤ 4, respectively. The yellow lines represent the relationship obtained by fitting all data using Reduced Major Axis regression, while the red line represents the fit using only the blue points. Each figure specifies the number of individuals per plot (NI) used in the simulation, along with the RMA-fitted slope and the corresponding *R^2^* value. All fitted results are significant at the *P* < 0.001 level.


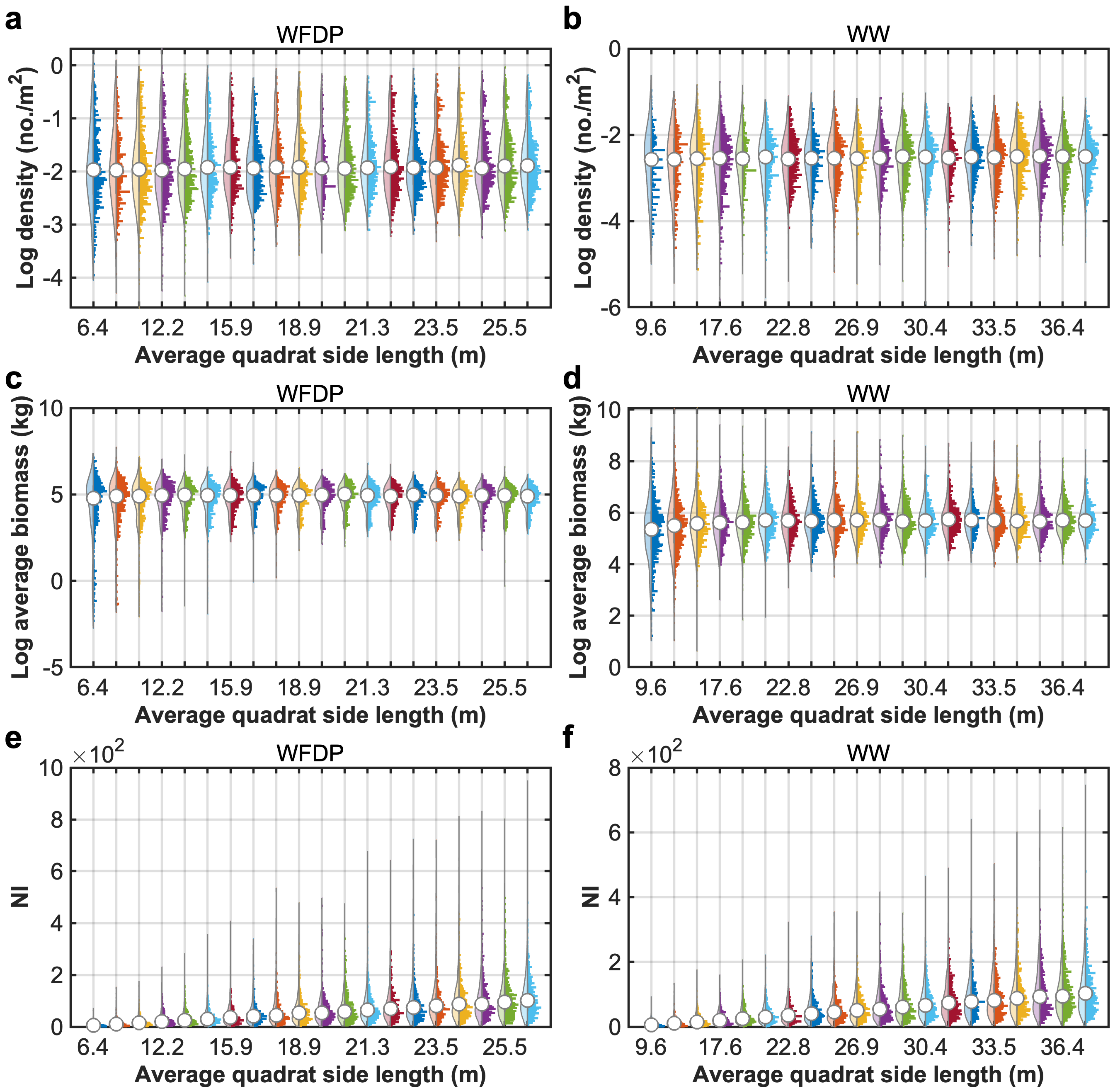


**Fig. S11.** **Simulation results for the quadrat-based method.** The distributions of log(density) (a, b), log(average biomass) (c, d), and the number of individuals per plot (e, f) obtained from simulations using the quadrat-based method, under varying quadrat side lengths, are illustrated. These distributions are illustrated using both probability density functions and histograms. The plot title indicates the dataset used in the simulation.


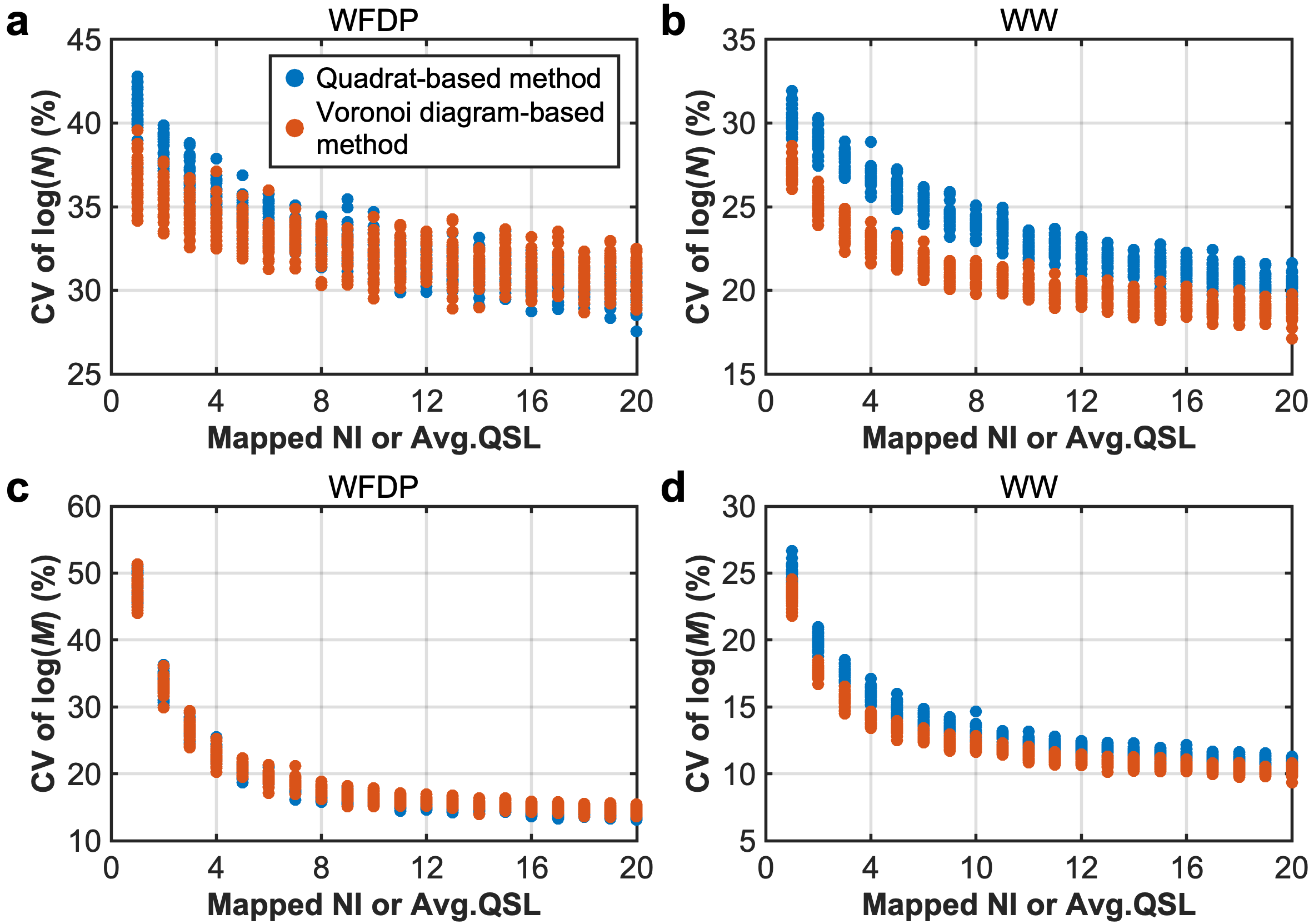


**Fig. S12.** **Comparisons between the Voronoi diagram-based and the quadrat-based method.** (a, b) Comparisons of the coefficient of variation for log(density) between the two methods, respectively. (c, d) The coefficient of variation for log(average biomass) was obtained using both methods. Red points represent results from the Voronoi diagram-based method, while blue points represent results from the quadrat-based method.


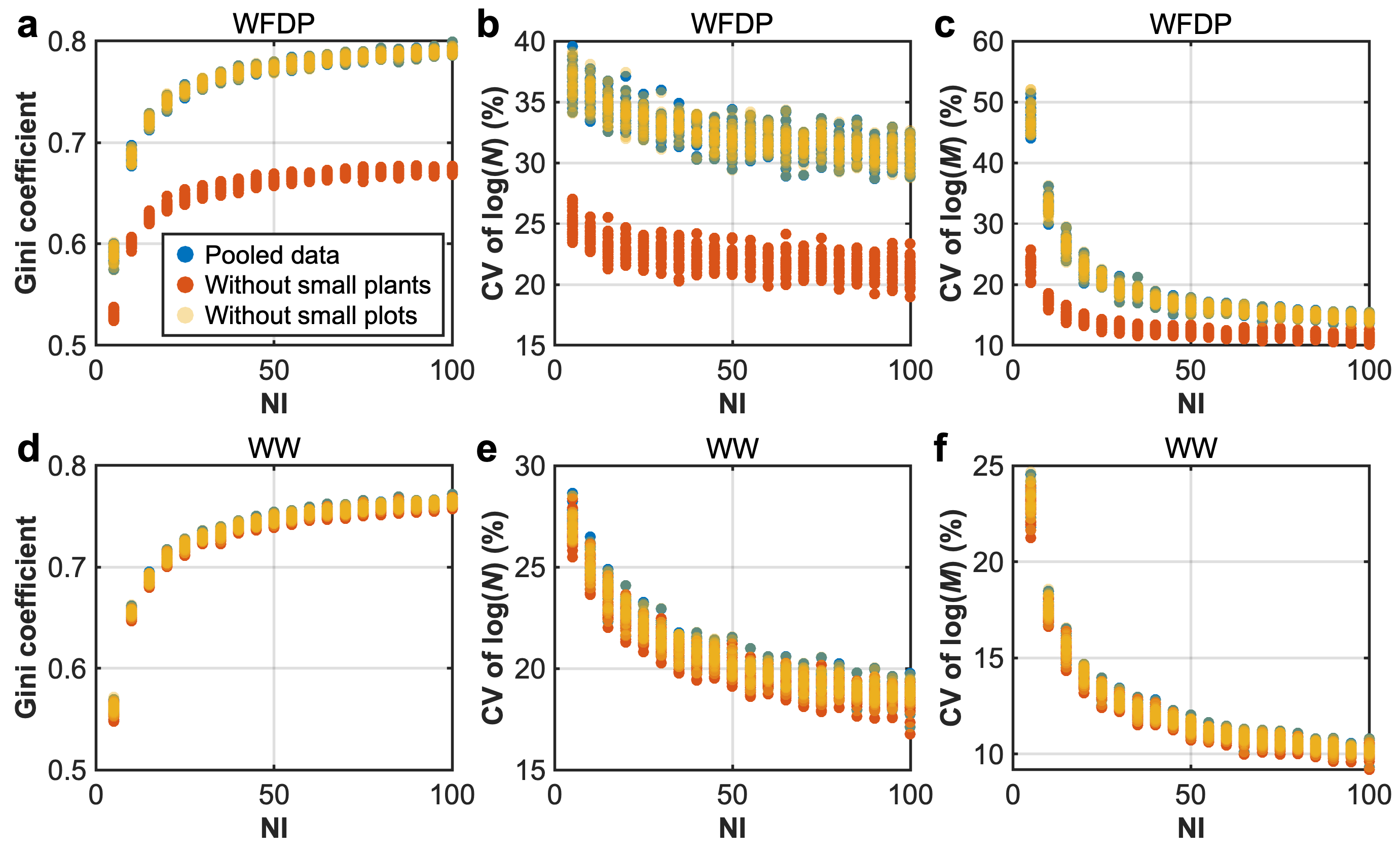


**Fig. S13.** **Comparisons of the Gini coefficient and the coefficient of variation (CV) for log(density) and log(average biomass) across three scenarios.** (a, d) Comparisons of the Gini coefficient for each plot across three scenarios. (b, e) Comparisons of CV for log(density) across three scenarios. (c, f) Comparisons of CV for log(average biomass) across three scenarios. Blue points represent results obtained using the Voronoi diagram-based method, red points represent results obtained in scenario 1, and yellow points correspond to simulation results in scenario 2. Due to the high degree of overlap among data points, some points may be obscured by others.
